# Effects of Malate Ringer's solution on myocardial injury in sepsis and enforcement effects of TPP@PAMAM-MR

**DOI:** 10.1186/s12967-022-03811-y

**Published:** 2022-12-13

**Authors:** Lei Tan, Han She, Jie Zheng, Xiaoyong Peng, Ningke Guo, Bindan Zhang, Yue Sun, Chunhua Ma, Shenglian Xu, Daiqin Bao, Yuanqun Zhou, Qinghui Li, Qingxiang Mao, Liangming Liu, Yi Hu, Tao Li

**Affiliations:** 1 Department of Anesthesiology, Daping Hospital, Army Medical University, Chongqing, 400042 China; 2 State Key Laboratory of Trauma, Burns and Combined Injury, Shock and Transfusion Department, Daping Hospital, Army Medical University, Chongqing, 400042 China; 3 School of Medicine, Chongqing University, Chongqing, 400044 China

**Keywords:** Sepsis, Myocardial injury, Mitochondrial function, Apoptosis

## Abstract

**Background:**

Myocardial dysfunction played a vital role in organ damage after sepsis. Fluid resuscitation was the essential treatment in which Lactate Ringer's solution (LR) was commonly used. Since LR easily led to hyperlactatemia, its resuscitation effect was limited. Malate Ringer's solution (MR) was a new resuscitation crystal liquid. Whether MR had a protective effect on myocardial injury in sepsis and the relevant mechanism need to be studied.

**Methods:**

The cecal ligation and puncture (CLP) inducing septic model and lipopolysaccharide (LPS) stimulating cardiomyocytes were used, and the cardiac function, the morphology and function of mitochondria were observed. The protective mechanism of MR on myocardial injury was explored by proteomics. Then the effects of TPP@PAMAM-MR, which consisted of the mitochondria- targeting polymer embodied malic acid, was further observed.

**Results:**

Compared with LR, MR resuscitation significantly prolonged survival time, improved the cardiac function, alleviated the damages of liver, kidney and lung following sepsis in rats. The proteomics of myocardial tissue showed that differently expressed proteins between MR and LR infusion involved oxidative phosphorylation, apoptosis. Further study found that MR decreased ROS, improved the mitochondrial morphology and function, and ultimately enhanced mitochondrial respiration and promoted ATP production. Moreover, MR infusion decreased the expression of apoptosis-related proteins and increased the expression of anti-apoptotic proteins. TPP@PAMAM@MA was a polymer formed by wrapping l-malic acid with poly amido amine (PAMAM) modified triphenylphosphine material. TPP@PAMAM-MR (TPP-MR), which was synthesized by replacing the l-malic acid of MR with TPP@PAMAM@MA, was more efficient in targeting myocardial mitochondria and was superior to MR in protecting the sepsis-inducing myocardial injury.

**Conclusion:**

MR was suitable for protecting myocardial injury after sepsis. The mechanism was related to MR improving the function and morphology of cardiomyocyte mitochondria and inhibiting cardiomyocyte apoptosis. The protective effect of TPP-MR was superior to MR.

**Supplementary Information:**

The online version contains supplementary material available at 10.1186/s12967-022-03811-y.

## Introduction

Sepsis is defined as a life-threatening organ dysfunction due to an overwhelming response to infection. About 31.5 million people worldwide suffer from sepsis yearly, and about 11 million people die of sepsis [1]. So far, sepsis is still a great challenge to human health. Cardiac dysfunction is one of the most severe complications of sepsis, which leads to poor outcome [2]. The mortality rate of sepsis patients suffering from cardiac dysfunction is as high as 40% [3–5]. Previous studies reported that the mechanism of myocardial injury after sepsis is mainly related to inflammation, autonomic dysfunction, and apoptosis [6, 7]. Corresponding treatment measures were also proposed, such as inflammatory factors scavenger and β receptor blockers, but the effect was not ideal. According to the guidelines for sepsis treatment [8], the fluid resuscitation is the essential method and at least 30 mL/kg of crystal solution is required to be administered intravenously within the first 3 h of resuscitation. Lactate Ringer's solution (LR) is commonly used for resuscitation in sepsis at present [9]. Although it could rapidly supplement the practical blood volume, the resuscitation effect of LR is limited because the lactate root (28 mmol/L) of LR could aggravate the accumulation of lactic acid in the body after sepsis and lead to hyperlactatemia, and the lower Na^+^ concentration (131 mmol/L) of LR easily leads to hyponatremia. Furthermore, the lower osmotic pressure of LR could exacerbate tissue edema when a large amount solution is infused. New solution is needed for the fluid resuscitation treatment of sepsis.

The heart requires a large amount of energy supply to maintain its physiological function [10]. Under physiological condition, fatty acids act as the major source of energy [11, 12]. Since cardiomyocytes only synthesize and store a small number of fatty acids, the required fatty acids mainly come from the circulation. However, previous studies found that metabolic model was changed from fatty acid oxidation to glucose oxidation after sepsis [13]. At same time, overexpression of inflammatory factors after sepsis, such as IL-1β, downregulated the expression of low-density lipoprotein receptors in cardiomyocytes, leading to inhibited lipid uptake in cardiomyocytes. Moreover, the expression of critical enzymes involved in fatty acid uptake, transport, and oxidation and transcription factors were inhibited, which led to an insufficient supply of mitochondrial oxidative phosphorylation substrate and further reduction of energy [14, 15]. So, effectively improving myocardial energy supply might play an essential role in alleviating myocardial injury in sepsis.

Malate Ringer's solution (MR) is a new type of resuscitation liquid which contains higher concentration of malate and bicarbonate ions but lower lactate ions as compared with LR. A study found that MR infusion could maintain stable circulation and pronounced therapeutic effect in patients with acute Ebola virus infection [16]. The other studies also showed that the effect of MR in the resuscitation for hemorrhagic shock was better than LR, and MR resuscitation was beneficial to organ function protection [17–19]. As a major component of MR, malic acid is an essential intermediate metabolite of the tricarboxylic acid cycle and the primary source of ATP in mitochondria. It was reported that malic acid could alleviate myocardial ischemia–reperfusion injury, enhance myocardial contractility, and reduce the expression of inflammatory factors [20, 21]. Whether MR has a protective effect on myocardial injury after sepsis and the underlying protection mechanism are still unclear.

l-malic acid is an essential component of MR. Previous studies found that the effect of l-malic acid was related to the amount of l-malic acid entering the mitochondria of cells [22]. Due to the loss in the process of entering mitochondria, l-malic acid might fail to achieve the desired effect. The targeted drug delivery system (DDS) was a kind of preparation that could introduce drugs into the body and improve the bioavailability and safety by controlling the release rate, time, and position of medicines [23, 24]. Dendrimer can encapsulate small molecule drugs through electrostatic force, van der Waals force, and H bond due to its unique properties (such as nanometer uniform hook size, high degree of branching, multivalent state, water solubility, available lumen, and convenient synthesis method) [25]. Common types of dendrimers include poly lysine (PLL), polypropylene imine (PPI), polyethylene imine (PEI), poly aryl ether, and poly amido amine (PAMAM). Compared with other dendrimers, PAMAM are generally spheroidal, highly branched, cascade polymers, the size and surface functional group can be finely controlled during synthesis. As an additional advantage, post-synthesis engineering of PAMAM dendrimers to produce alternative, often open, structural architectures is also possible. Such PAMAM nanoarchitectures allow for improved drug entrapment and delivery through increasing the capacity of drug pay-load which can be physically carried internally within the voids of the dendrimer [26]. Thus, we wrapped l-malic acid with TPP-PEG-PAMAM, a material targeting mitochondria, to improve the bioavailability of l-malic acid. TPP@PAMAM@MA replaced the l-malic acid in MR to get the new resuscitation solution TPP@PAMAM-MR (TPP-MR).

Therefore, in present study, the cecal ligation and puncture (CLP) rats model and lipopolysaccharide (LPS)-stimulated cardiomyocytes were applied to explore the protective effect and mechanism of MR on myocardial function in sepsis. The therapeutic effects of MR and TPP-MR were compared.

## Materials and methods

### Ethical approval of the study protocol

The study protocol was approved by the Research Council and Animal Care and Use Committee of Research Institute of Surgery, Daping Hospital, Army Medical University (Chongqing, China, approval number SYXK-20170002). The investigation conformed to the Guide for the Care and Use of Laboratory Animals published by the US National Institutes of Health (8th edition, 2011, National Institutes of Health, Bethesda, Md).

### Reagents

Malate Ringer's solution was purchased from B. Braun (Melsungen AG, Germany). Lactate Ringer's solution was purchased from Sichuan Kelun Pharmaceutical (Sichuan, China). Sodium chloride, potassium chloride, calcium chloride, magnesium chloride and sodium acetate were purchased from Macklin Biochemical Co Ltd (Shanghai, China). MitoTracker Deep Red (M22426) was purchased from Invitrogen (Carlsbad, CA, USA). Malic acid detection kit was purchased from Cell Biolabs, Inc (San Diego, USA). The JC-1 fluorescent probes for mitochondrial membrane potential (△Ψm) detection (C2006), and DCFH-DA fluorescent probes for reactive oxygen species (ROS) detection (S0033) were purchased from Beyotime Biotechnology (Shanghai, China). In situ Cell Death Detection Kit (TUNEL) (11767291910) was purchased from Roche (Basel, Switzerland). ELISA kits of Interleukin-1β (IL-1β) (Cat.E-EL-R0012c), Interleukin-6 (IL-6) (Cat.E-EL-R0015c), Tumor necrosis factor-α (TNF-α) (Cat.E-EL-R2856c) were purchased from Elabscience company (Wuhan, China). OCR (mitochondrial respiration detection) kit, lipopolysaccharide (LPS) was purchased from Sigma (St. Louis, Missouri, USA). Cytc (11940s), Caspase-3 (9662s), Bax (14796s), Bcl-xl (2764s) antibodies were purchased from Cell Signaling Technology (Danvers, Massachusetts, USA). Bcl-2 (GTX100064) antibody was purchased from GeneTex (San Antonio, TX, USA). Triphenyl phosphine (TPP) (T84409), PAMAM (412449) was purchased from Sigma (St. Louis, Missouri, USA).

### The cecal ligation and puncture (CLP) septic model and groups

Adult SD rats (weight 200–220 g, provided by the experimental animal center of Daping Hospital of Army Medical University) were anesthetized by intraperitoneal injection of sodium pentobarbital (45 mg/kg). SD rats fasted for 8 h before the operation and drank water freely. According to a previous study [27], cecal ligation and puncture (CLP) was used to replicate the sepsis model in rats. The abdomen was disinfected routinely, and the skin was cut straight along the abdominal to expose the cecum. Ligated the cecum at 0.7 cm away from the distal end (needle diameter 1.5 mm), pushed the feces gently toward the distal cecum, perforated the end of the cecum with a conical device, and the feces naturally flowed into the abdominal cavity. Then relocated the cecum, sutured the wound layer by layer, and injected sterile normal saline intraperitoneally at 2 mL/100 g. Placed the animals back in the cage where access to water but not food was available. Twelve hours after the operation, the femoral artery was intubated to monitor the mean arterial blood pressure (MAP). The CLP model was established successfully when the MAP decreased by 30% or more.

According to the guideline of sepsis treatment [3], the sham group was given a laparotomy but not ligated or perforated, the sepsis group only received the CLP modeling, and the rats were infused with Lactate Ringer's solution (LR) at 12 h after CLP operation in the LR group. At same time, Cefuroxime sodium (100 mg/kg) and vasoactive drug dopamine (DA) were given to ensure that the MAP was above 70 mm Hg and the central venous pressure was maintained at 8 mmHg. For the animals in MR the group, LR was replaced by MR (35 mL/kg) at a constant rate of 2.5 mL/h. For the animals in the TPP-MR group, MR was replaced by TPP-MR at a constant rate of 2.5 mL/h. After the infusion, the blood vessels were ligated, and the incision was sutured. Then the mean survival time and the survival rate of rats within 24 h were recorded, and the following experiment was carried out.

### Treatment of cardiomyocytes

The neonatal rat cardiomyocytes (NRCMs) were extracted according to the reference [28] and cultured in DMEM supplemented with 10% FBS in a humidified, 5% CO_2_/95% air atmosphere at 37 °C. The H9C2 cardiomyocytes were obtained from the American Type Culture Collection (ATCC, Manassas, VA, United States) and cultured in DMEM supplemented with 10% FBS in a humidified, 5% CO_2_/95% air atmosphere at 37 °C. When the cardiomyocytes covered more than 70% of the culture dish, cells were randomly received DMEM (Normal group), [29] 1 µg/mL LPS (LPS group), 1 µg/mL LPS + 1% volume [30] of LR (LR group), and 1 µg/mL LPS + 1% volume of MR solution (MR group) for 12 h, respectively. After the treatment, the cells were collected for related experiments.

### Cardiac function measurement

The cardiac output (CO) value and heart rate (HR) were recorded by cardiac output meter (Cardiomax III, USA) [31]. The cardiac index (CI) and stroke index (SI) were calculated by the following formula: CI = CO ÷ S, S = K × W^2/3^ (cm^2^), K = 9.1, W = weight (g), SI = CI ÷ HR. Arterial and venous blood gas were analyzed as following: partial pressure of oxygen (pO2), hemoglobin (Hb), arterial oxygen saturation (SaO_2_), and venous oxygen saturation (SvO_2_). Tissue oxygen supply (DO_2_ = CI × 13.4 × Hb × SaO_2_) and tissue oxygen consumption [VO2 = CI × 13.4 × Hb × (SaO_2_-SvO_2_)] were also calculated [32].

### Proteomics analysis

The left ventricular tissues of MR group and LR group (n = 3/group) were collected for proteomics analysis. For each sample, the total peptides (1 µg) were separated and analyzed with a nano-UPLC (EASY-nLC1200) coupled to a Q Exactive HFX Orbitrap instrument (Thermo Fisher Scientific) with a nano-electrospray ion source. Separation was performed using a reversed-phase column. Mobile phases were H_2_O with 0.1% FA, 2% ACN (phase A) and 80% ACN, 0.1% FA (phase B). Separation was executed with a 90 min gradient at 300 nL/min flow rate. Gradient B: 2–5% for 2 min, 5–22% for 68 min, 22–45% for 16 min, 45–95% for 2 min, 95% for 2 min. Data dependent acquisition (DDA) was performed in profile and positive mode with Orbitrap analyzer at a resolution of 120,000 (@200 m/z) and m/z range of 350–1600 for MS1; For MS2, the resolution was set to 45k with a fixed first mass of 110 m/z. The automatic gain control (AGC) target for MS1 was set to 3E6 with max IT 30 ms, and 1E5 for MS2 with max IT 96 ms. The top 20 most intense ions were fragmented by HCD with normalized collision energy (NCE) of 32% and isolation window of 0.7 m/z. The dynamic exclusion time window was 45 s, single peak and peaks exceeding 6 were excluded from the DDA procedure. Vendor’s raw MS files were processed using Proteome Discoverer (PD) software (Version 2.4.0.305) and the built-in Sequest HT search engine. MS spectra lists were searched in their species-level UniProt FASTA databases (uniprot-Rattus + norvegicus-10116-2020-10. fasta) with Carbamidomethyl [C], TMT pro (K) and TMT pro (N-term) as a fixed modification and Oxidation (M) and Acetyl (Protein N-term) as variable modifications. Trypsin was used as proteases. A maximum of 2 missed cleavage(s) was allowed. The false discovery rate (FDR) was set to 0.01 for both PSM and peptide levels. Peptide identification was performed with an initial precursor mass deviation of up to 10 ppm and a fragment mass deviation of 0.02 Da. Unique peptide and Razor peptide were used for protein quantification and total peptide amount for normalization. All the other parameters were reserved as default.

### Detection of malic acid content in myocardial tissue

The content of malic acid in myocardial tissue was measured by malic acid determination kit (fluorescence method). The 20 mg tissues from the left ventricle (n = 3/group) were collected and cut into pieces. Put the tissues into the 1.5 mL EP tube, added 500 µL distilled water for homogenization for 30 min, centrifuged at 10,000r at room temperature for 10 min. Then 50 µL supernatant containing malic acid plus 50 µL detection working solution were added into 96 well micro titer blackboard, and the mixed liquids were incubated at room temperature for 45–60 min under light conditions. Finally, the values at the excitation wavelength of 560 nm and the emission wavelength of 590 nm were recorded by the microplate fluorometer.

### Mitochondrial respiration (OCR) assay

A total volume of 100 µL cardiomyocyte suspension was inoculated into each well of the XF24 cell culture plate except the background correction hole [33], and the cell suspension density was 10K cells/100 μL/well. After the cells adhered to the wall, 150 µL high sugar medium was added into each well. Following 1 µg/mL LPS stimulation for 12 h, cells in the LR group were incubation with 1% LR at same time. And the cells in the MR group was incubation with 1% volume MR. Put the cell culture plate into the 37 °C CO_2_ cell incubator for 12 h, added 1 mL XF calibration solution to each well of the hydration plate, and put the whole probe plate into the 37 °C CO_2_ free cell incubator for hydration overnight. Then the cells in each well of the cardiomyocyte culture plate were washed twice, and 500 mL detection solution was guaranteed for each well. The cell culture plate was put into the CO_2_ free cell incubator at 37 °C for 60 min, waiting for the machine detection. The XFe24 probe plates were processed according to each experimental scheme, and the machine detection was carried out for the XF Cell Mito Stress Test Kit of cardiomyocytes.

### Mitochondrial membrane potential measurement

Cardiomyocytes were inoculated into confocal petri dishes. Added ultrapure water to 50 µL 200 × JC-1 to 8 mL and shook them violently to mix thoroughly, and added 2 mL dyeing buffer (5x) to prepare JC-1 dyeing working solution. Added 0.5 mL JC-1 working solution into cardiomyocytes in each petri dish, and incubated the cells in the cell incubator at 37 °C for 20 min. Then washed the cells twice with JC-1 staining buffer (1x), added the cell culture solution, and observed the expression of JC-1 under the laser confocal microscope. The cells in a normal state should show red fluorescence, and those with a loss of mitochondrial membrane potential should show green fluorescence.

### Reactive oxygen detection

Cardiomyocytes were inoculated into confocal petri dishes. DCFH-DA was diluted with serum-free medium to 1:1000 (ROS kit contains DCFH-DA probe and reactive oxygen species positive control) so that the final concentration was 10 µmol/L. Added the diluted DCFH-DA into the culture dish and placed it in the cell incubator at 37 °C for 20 min. To make the probe entirely in contact with the cells, shook the culture dish in parallel every 5 min to fully remove the redundant DCFH-DA, washed the cells with a serum-free medium 3 times, and finally observed under the laser confocal microscope.

### The morphology of mitochondria in myocardial tissue was observed by transmission electron microscope

Fresh heart tissues were taken from each group and were quickly fixed with arsenate buffer containing 2.5% glutaraldehyde for 24 h (pH = 7.4, 4 °C). Washed the tissues 3 times with PBS for 10 min, fixed them with 1% osmium tetroxide at room temperature for 2 h, and then dehydrated them in graded ethanol (65%,70%,75%,80%, 95% for 10 min successively). Subsequently, the heart tissues were incubated with TERT butylamine for 10 min, then dried with carbon dioxide, stained with uranyl acetate or lead citrate, and coated with gold (Au) with a sputtering ion coater. Finally, the morphology of mitochondria was observed and imaged by transmission electron microscope (H-7500, Hitachi, Japan).

### The morphology of mitochondria was observed by confocal microscope

The cardiomyocytes were inoculated into the confocal culture dish. After 12 h treatment according to each group, cardiomyocytes were washed with PBS at 37 °C, and the configured Mito-tracker (1:10,000) was added. After incubation at 37 °C for 30 min, they were washed with PBS at 37 °C, and 1 mL serum-free medium was added to the culture dish. Then, the morphology of mitochondria in cardiomyocytes were observed by confocal microscope.

### Isolation of cardiomyocytes and observation of mitochondrial morphology in rats

After draining the congestion, the fresh rat heart was immersed in the digestive juice to make the heart tissue soft [34]. Then the heat was removed into liquid B (25 mL calcium-free Steiner's solution + 25mgBSA + 50 μM Ca^2+^), and the outer membrane was torn off with microscopic tweezers. Blew the heart tissue slowly with a pasteurized tube until the cardiomyocytes dispersed into liquid B. The remaining connective tissue was removed by filtration with a 200-mesh sieve and the filtered liquid was collected. The digested cardiomyocytes were obtained after centrifugation at room temperature (500r, 30 s). After dyeing with Fura2-AM (Calcium ion fluorescence probe), 10 µL cell suspension was transferred into the stimulation bath, let it stand to make the cells adhere to the wall. Added the taishi solution (1.8 mMCa^2+^), observed the interested cells under the 40× microscope, and counted the number of mitochondria of a single cardiomyocyte.

### Synthesis of TPP@PAMAM@MA

Triphenyl phosphine [35, 36] (TPP, 10 mmol) and 6-bromohexanoic acid (10.5 mmol) were dissolved in anhydrous acetonitrile and refluxed for 16 h under the protection of nitrogen. The reactant was recrystallized to obtain TPP-COOH. Then, TPP-COOH (2 mmol), *N*,*N*-dicyclohexylcarbodiimide (2.4 mmol) and *N*-monohydroxy succinimide (NHS, 2. 4 mmo1) were mixed in 10 mL anhydrous DMSO and reacted at room temperature for 12 h, then PEG (1 mmol) was added to continue the reaction at room temperature. Following 12 h, the reaction solution was transferred to a dialysis bag (with a cut-off molecular weight of 1000), and dialyzed with DMSO for 24 h and deionized water for 48 h. The dialysate was freeze-dried to obtain intermediate products (TPP-PEG). Next, TPP-PEG (1 mmol), *N*,*N*-dicyclohexylcarbodiimide (1.2 mmol) and *N*-monohydroxy succinimide (NHS, 1.2 mmo1) were mixed in 5 mL anhydrous DMSO and reacted at room temperature for 24 h, then polyamidoamine [37] (PAMAM, 3 mmol) was added to continue the reaction at room temperature. After 24 h, the reaction solution was transferred to a dialysis bag (with a cut-off molecular weight of 2000), and dialyzed with DMSO for 24 h and deionized water for 48 h. The dialysate was freeze-dried to obtain intermediate products (TPP-PEG- PAMAM). Finally, equivalent malic acid was mixed with TPP-PEG-PAMAM solution by gentle pipetting. The mixture was vortexed for 30 s, and then kept still at room temperature for 30 min to form a TPP-PEG-PAMAM-malic acid polymer [38, 39]. Hereinafter referred to as TPP@PAMAM@MA. DMSO-d was used as solvent, TPP@PAMAM@MA was identified by 1HNMR, particle size distribution, zeta potential, and electron microscopic.

### Effects of TPP@PAMAM@MA on organ toxicity and H9C2 cell viability

TPP@PAMAM@MA was dissolved in sterilized water and prepared into a 10 mg/mL solution. After 24 h treated with 30 mg/kg TPP@PAMAM@MA intravenous injection, the heart, liver and kidney tissues of septic rats were collected for HE staining. About 5000 cardiomyocytes (H9C2)/well were cultured in 96 well plates, then TPP@PAMAM@MA (1 µg/mL) was added for 15 min at 37 ℃. Cell counting reagent SF were used to determine the survival rate of the cells.

### Preparation of TPP@PAMAM@MA

TPP@PAMAM@MA was synthesized by nanomaterial synthesis technology. MR was prepared in the following proportions: sodium chloride 6.799 g, potassium chloride 0.2984 g, calcium chloride 0.3675 g, magnesium chloride 0.2033 g, sodium acetate 3.266 g, l-malic acid 0.671 g, sodium hydroxide 0.2 g, constant volume configuration to 1000 mL with injection water. The l-malic acid in MR was replaced by TPP@PAMAM@MA to get the new resuscitation solution TPP@PAMAM-MR (TPP-MR). The dose needed by TPP-MR was calculated based on the entrapment rate and drug loading.

### Sample preparation and analysis TPP@PAMAM@MA

TPP@PAMAM-MR is different from MR. TPP@PAMAM-MR is to replace l-malic acid in MR with TPP@PAMAM@MA, which was a polymer formed by wrapping l-malic acid with poly amido amine (PAMAM) modified triphenylphosphine material. We measured the drug loading and peak loading rate of TPP@PAMAM@MA, 24.27% and 80.09%. According to the drug load and peak loading rate, replace the l-malic acid component in MR with TPP@PAMAM @MA, l-malic acid with equal mass, and prepared into TPP-MR. Sixty SD rats were randomly divided into MR and TPP-MR groups according to 10 time points, with 30 in each group. TPP-MR and MR were infused intravenously at a dose of 35 mL/kg in each group. Blood was collected from the abdominal main vein at 10 min, 30 min, 1 h, 2 h, 4 h, 8 h, 24 h, 36 h, 48 h and 72 h after administration, and the mice were killed immediately after dissection. 200 μL plasma and control plasma were added 400 μL methanol, was vortexed for 1 min, and was centrifuged at 4 °C for 10 min at 12,000 r/min, the supernatant was collected, and then was freeze dried, 100 μL methanol was added to the residue redissolved, and was centrifuged at 12,000 r/min for 10 min, the supernatant was collected, 10 μL supernatant was injected into LC–MS/MS system for analysis.

### Statistical analysis

SPSS20.0 (SPSS Inc., Chicago, IL, USA) was used for statistical analysis. The data were represented as means ± SD. An independent sample t-test was used to analyze the difference between the two groups. One-way analysis of variance (ANOVA) and post hoc test (S-N-K/LSD) were used for more than two groups. P < 0.05 was considered statistically significant.

## Results

### Protective effects of MR on sepsis

#### Effect of MR on cardiac function in septic rats

**Fig. 1:**
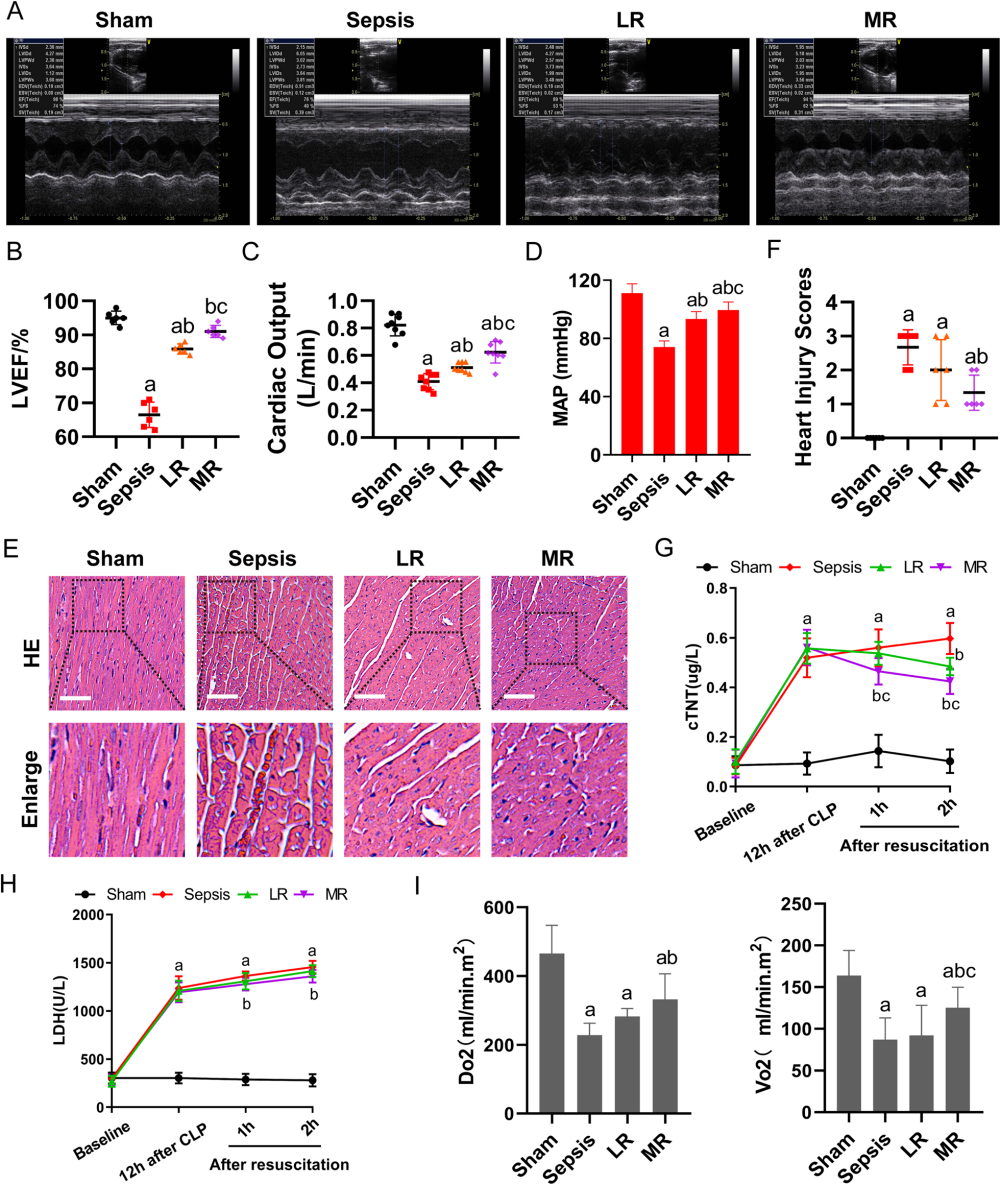
Effects of MR on cardiac function in septic rats. Cardiac function including **A** echocardiograms (n = 6/group), **B** LVEF (n = 6/group) and **C** cardiac output (n = 8/group) were measured 2 h after resuscitation treatment. **D** Mean arterial pressure measurement of the septic rats (n = 8/group). **E**, **F** HE stains of cardiac pathological sections and heart injury scores (bar, 40 µm, n = 8/group). The expressions of **G** cardiac troponin T and **H** lactate dehydrogenase in venous blood (n = 8/group). **I** Detection of oxygen supply and consumption of the septic rats (n = 8/group). a: P < 0.05 compared with the sham or normal group, b: P < 0.05 compared with the sepsis or LPS group, c: P < 0.05 compared with the LR group

The echocardiography was used to assess cardiac function. The results showed that the LVEF of septic rats was significantly decreased. LR infusion slightly improved LVEF. After MR infusion, LVEF was significantly improved, the increase ratio was 7.1% as compared with the LR group (Fig. 1A, B). In addition, the sepsis led to a significant decrease in cardiac output (CO), cardiac index (CI) and stroke index (SI). LR infusion slightly improved cardiac function. After MR infusion, CO, CI and SI were significantly improved, the increase ratio was 21.7%, 22.5%, and 89.3% respectively as compared with the LR group (p < 0.05) (Fig. 1C and Additional file 1: Fig. S1A, B). The MAP was decreased to 60 mmHg in septic rats. The MAP was raised to 80 mmHg after LR infusion. After MR infusion, the MAP was increased by 6.6% as compared with the LR group (P < 0.05) (Fig. 1D).

HE staining of cardiac pathological sections showed that the myocardial fiber structure was orderly and the interstitial tissue was rare in normal myocardial tissue. After sepsis, myocardial cells were significantly swollen with vacuole formation, myocardial fibers were arranged disorderly, and the interstitial space was widened. The LR infusion only slightly alleviated sepsis-induced changes of cardiac histology. In MR group, the interstitial edema of myocardial cells was significantly relieved, the interstitial space was reduced, and the disorder of myocardial fiber arrangement was significantly restored (Fig. 1E). Pathological score according to the reference [40] (Fig. 1F).

The cardiac injury markers cardiac troponin T(cTnT) and lactate dehydrogenase (LDH) were increased significantly after sepsis, indicating that the myocardial function was impaired. After LR infusion, the level of cTnT was decreased slightly, while the level of LDH was maintained at high level. Compared with the LR group, the levels of cTnT and LDH were decreased significantly in the MR group, indicating that myocardial injury was improved considerably (Fig. 1G, H).

The oxygen delivery and consumption of septic rats were decreased significantly. After LR infusion, oxygen delivery and consumption were slightly improved. After MR infusion, the oxygen delivery and consumption increased significantly, increasing by 17.5% and 36%, respectively as compared with the LR group (p < 0.05) (Fig. 1I). These results suggested that MR could substantially improve the cardiac function of septic rats.

#### Protective effects of MR on liver and kidney function and survival rate in septic rats

**Fig. 2:**
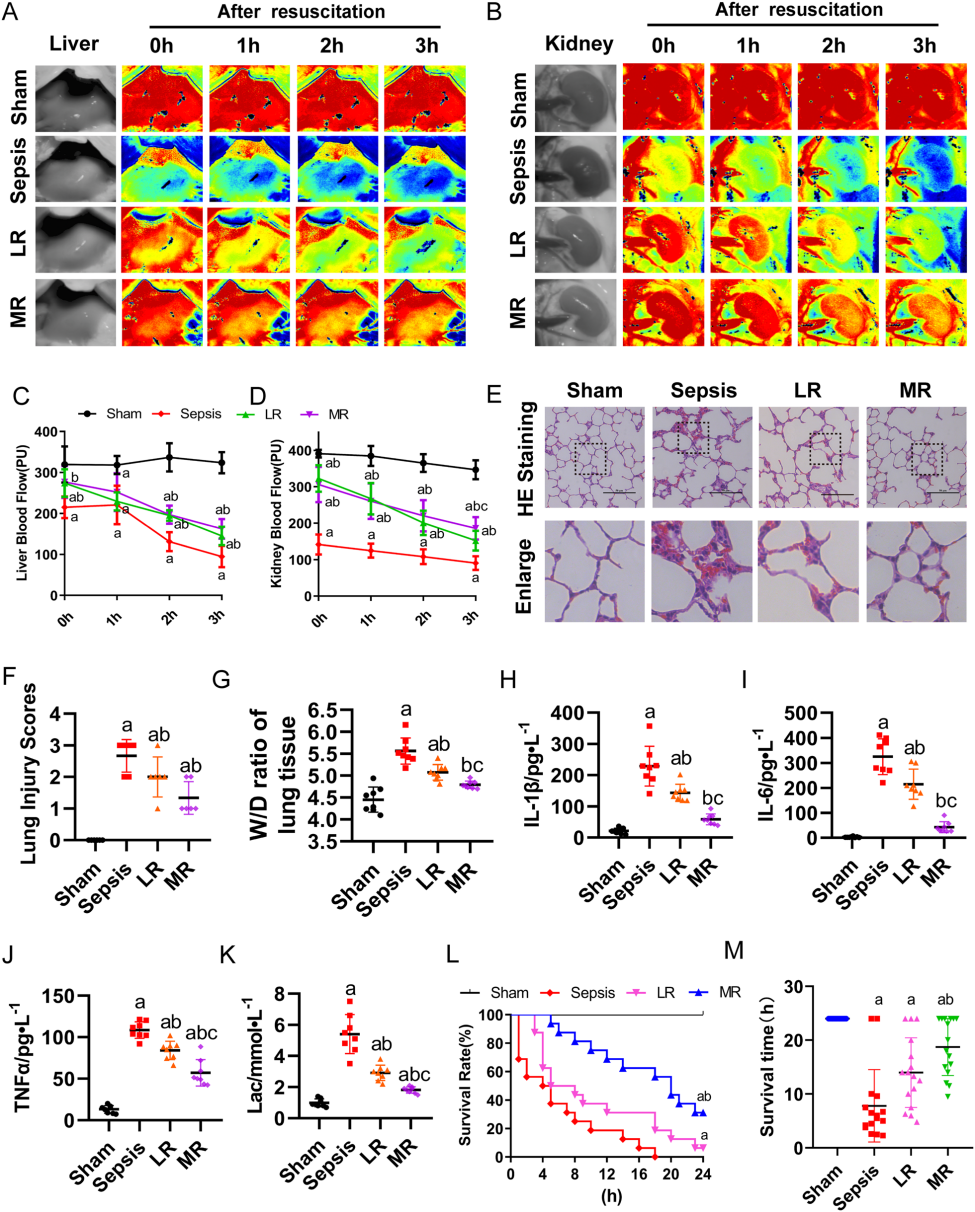
The effect of MR on the function of the liver, kidney, and the survival rate. **A**, **C** The liver blood flow at 0 h, 1 h, 2 h and 3 h after the resuscitation (n = 8/group). **B**, **D** The kidney blood flow at 0 h, 1 h, 2 h, and 3 h after the resuscitation (n = 8/group). **E** Representative images of pulmonary histology and **F** lung injury scores were evaluated under different conditions (bar, 50 µm, n = 6/group). **G** The wet/dry weight ratio of lung tissue at 2 h after resuscitation (n = 8/group). The level of **H** IL-1β, **I** IL-6 and **J** TNF-α in venous blood (n = 8/group). **G** Serum lactic acid level (n = 8/group). **L**, **M** Survival rate and survival time were observed in different groups (n = 16/group). a: P < 0.05 compared with the sham or normal group, b: P < 0.05 compared with the sepsis or LPS group, c: P < 0.05 compared with the LR group

The blood flow of liver and kidney were observed by laser blood flow meter at 0 h, 1 h, 2 h, and 3 h after the resuscitation. Sepsis led to the decrease of the hepatic and renal blood flow significantly. LR infusion improved the blood flows of the liver and kidney for the first 2 h of resuscitation, while they were decreased after 3 h of resuscitation. By contrast, MR infusion improved the hepatic and renal blood flows significantly for 3 h of resuscitation, the hepatic blood flows were increased by 28.4%, 14.5%, 50.4% and 92.3% respectively after MR resuscitation 0 h, 1 h, 2 h, and 3 h, and the renal blood flows were increased by 117%, 109.7%, 103.7% and 105.6%, respectively (Fig. 2A–D). The results of lung HE staining showed that the alveoli in the sham group were integrated with was no obvious exudation. Notably, disordered alveolar structure was found in the sepsis group, with pulmonary interstitial edema, and a large number of erythrocyte and inflammatory cells in the alveolar cavity. Treatment with MR could alleviate lung injury, improve alveolar structure, decrease interstitium edema, and reduce erythrocytes and inflammatory cells infiltration (Fig. 2E). Pathological scoring according to the reference [40] (Fig. 2F). In addition, the wet/dry (W/D) ratio of lung tissue increased significantly after sepsis. After MR infusion, W/D ratio was significantly reduced as compared with the LR group (Fig. 2G). The serum levels of IL-1β, IL-6 and TNF-α in septic rats were significantly increased. LR infusion could decrease the levels of them. After MR infusion, the levels of IL-6, IL-1β, and TNF-α in septic rats decreased by 59.1%, 75.5%, and 32.1% as compared with the LR group (Fig. 2H, J). The liver and kidney function index (ALT, AST, Crea, and Urea) were also detected. MR infusion significantly alleviated the damages of liver and kidney function (Additional file 2: Fig. S2A–D). The level of blood lactic acid increased significantly in sepsis and LR groups. After MR infusion, the levels of the lactic acid in sepsis rats decreased by 37.8% compared with the LR group (Fig. 2K).

None of the rats was survived to 24 h in the sepsis group. The survival time of the LR group was slightly improved than that of sepsis group, and the 24-h survival rate was 6.25%. The survival time in MR group was significantly prolonged to 17.3 ± 13.56 h, and the 24 h survival rate was 31.3% (Fig. 2L, M). The above results indicated that MR was beneficial to septic rats as it improved the function of liver and kidney, reduced pulmonary edema, inhibited the release of inflammatory factors, and prolonged the survival time.

### The mechanisms of MR protecting cardiac function in sepsis

#### The proteomics in myocardium after septic rats

**Fig. 3:**
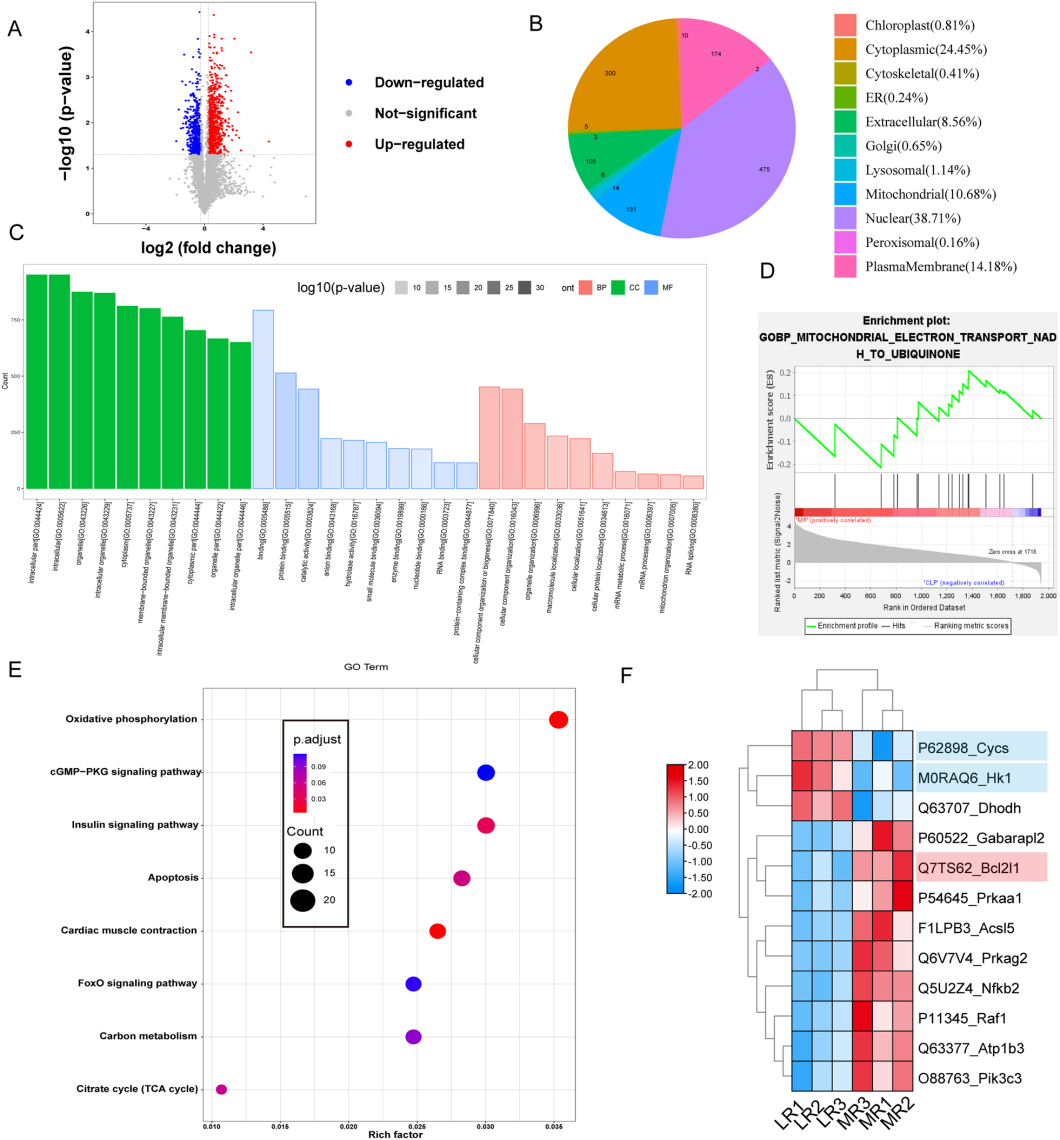
Proteomics analysis between LR and MR group. **A** Volcano plot of the differentially expressed proteins between the MR group and the LR group, n = 3/group. **B** Subcellular organelle localization of differentially expressed proteins. **C** GO enrichment analysis. **D** GSEA enrichment analysis. **E** KEGG bubble diagram. Each bubble represents a path. **F** Heat map of differentially expressed proteins

The heart tissues from the LR group and MR group were collected for proteomic detection. The screening conditions for differential proteins were unique peptide ≥ 1, foldchange > 1.2 or < 0.83, and p-value < 0.05. Volcano analysis showed 1227 significantly different proteins in cardiac tissues between MR and LR groups. There were 464 proteins down regulated and 763 proteins up regulated (Fig. 3A). The subcellular localization of the differential proteins showed that most of them were located in the nucleus, followed in the cytoplasm and mitochondria (Fig. 3B). To analysis of differential proteins, the DAVID database was applied. The results showed that the differential proteins were mainly involved in cell localization, protein localization, organic nitrogen metabolism, and other biological processes. Protein binding, nucleotide binding, catalytic activity, and other molecular functions (Fig. 3C). The enrichment analysis found that differential proteins had a significant effect on mitochondrial electron transport (Fig. 3D). In addition, through the pathway enrichment of differential proteins in the KEGG database [41], it was found that cell oxidative phosphorylation, apoptosis, and myocardial contraction were involved, suggesting that MR may regulate the mitochondrial function and apoptosis of cardiomyocytes (Fig. 3E). The expression of proapoptotic protein Cycs and glycolysis-related protein HK1 were significantly decreased, and the anti-apoptotic protein Bcl2l1 were increased considerably after MR treatment (Fig. 3F).

#### Protective effect of MR on myocardial mitochondrial function in sepsis

**Fig. 4:**
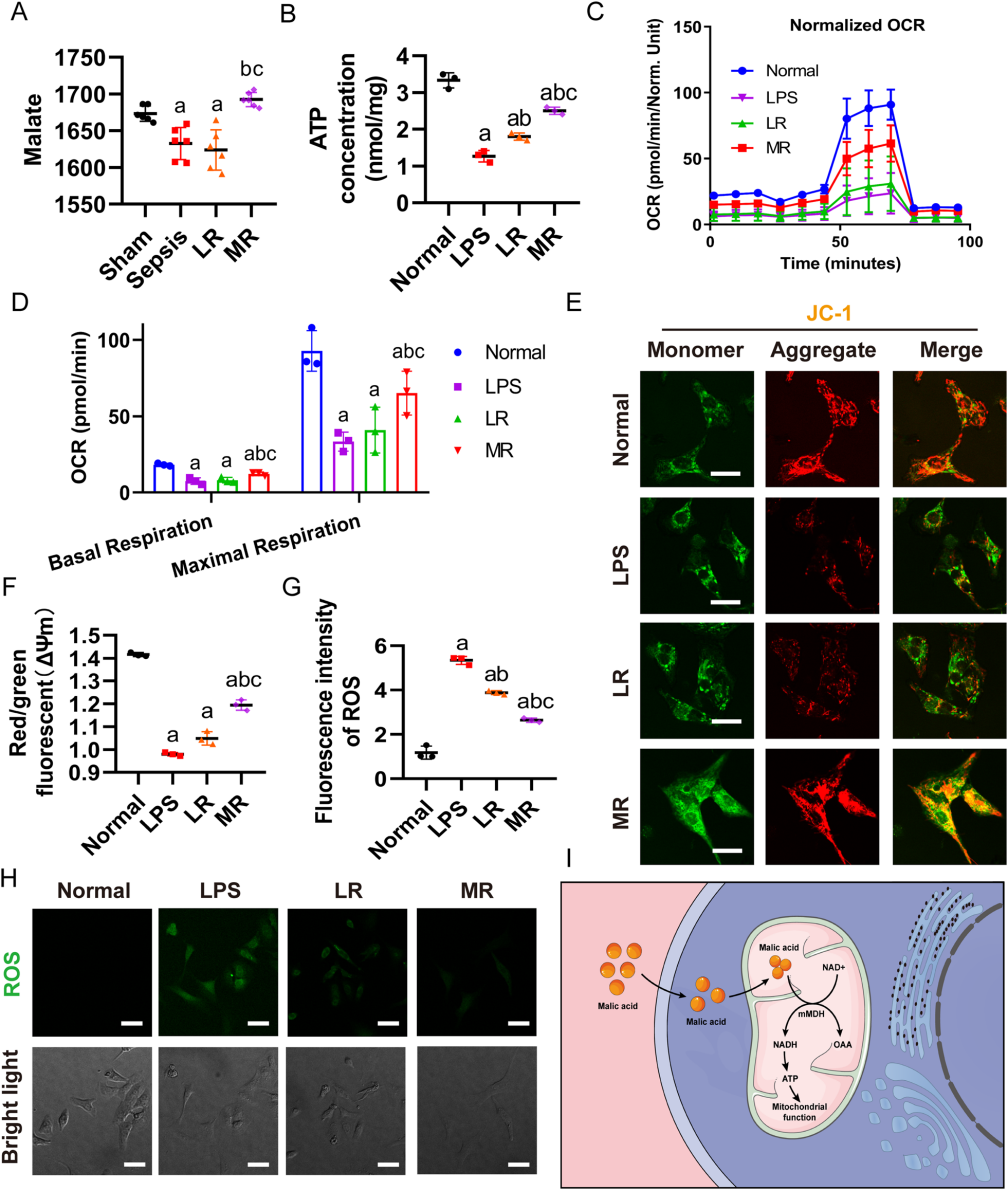
Mitochondrial function of cardiomyocytes after sepsis. **A** The content of malic acid in heart tissue was detected 2 h after resuscitation, n = 6/group. **B** Changes in the ATP level (3 independent experiments). **C**, **D** Mitochondrial maximum respiratory rate assay in NRCMs (3 independent experiments). **E** The mitochondrial membrane potential of NRCMs was observed by confocal microscopy (bar, 30 µm, 3 independent experiments). **F** Red/green fluorescent (Δψm) quantitative analysis in NRCMs. **G** The content of reactive oxygen species in NRCMs was observed by confocal microscopy (bar, 50 µm, 3 independent experiments). **H** ROS fluorescence quantitative analysis in NRCMs. **I** The mechanism of MR protecting mitochondria function of cardiomyocytes. a: P < 0.05 compared with the sham or normal group, b: P < 0.05 compared with the sepsis or LPS group, c: P < 0.05 compared with the LR group

The malic acid level was decreased in the heart tissues after sepsis, and LR infusion did not increase the level of malic acid. The malic acid content in MR group was increased significantly by 4% as compared with the LR group (p < 0.05) (Fig. 4A). The neonatal rat cardiomyocytes (NRCMs) were used to observe the effects of malic acid which was identified by cardiac troponin T (Additional file 3: Fig. S3). To explore whether cardiomyocyte mitochondria can be improved after increasing malic acid content, LPS stimulated neonatal rat cardiomyocytes (NRCMs) were used to observe the mitochondrial respiration (OCR) by Agilent Hippocampal XF technique. The results showed that LPS stimulation significantly reduced OCR of NRCMs, which was improved considerably by MR infusion but not LR. In addition, MR infusion increased the production of ATP significantly as compared LR infusion (Fig. 4B–D). The same results were also observed in H9C2 cells (Additional file 4: Fig. S4A–C). The mitochondrial membrane potential of JC-1 and the level of ROS marked by DCGH-DA in each group were further observed. After LPS stimulation, the mitochondrial membrane potential of NRCMs were decreased significantly, and the fluorescence intensity of ROS was increased significantly. Following LR infusion, the mitochondrial membrane potential and ROS fluorescence intensity of NRCMs did not ameliorate as compared with the sepsis group. While MR infusion could antagonize the sepsis-induced changes of mitochondrial membrane potential and ROS fluorescence intensity, indicating that MR infusion reduced sepsis-induced damage of mitochondrial function in cardiomyocytes (Fig. 4E–H). The trend was the same in H9C2 cells (Additional file 4: Fig. S4D, E). The mechanisms may be related to the exogenous malic acid in MR entering into mitochondria through the malic acid aspartate shuttle, and then undergoing the reduction reaction by malate dehydrogenase which improves the production of ATP (Fig. 4G).

#### Effect of MR on mitochondrial morphology

**Fig. 5:**
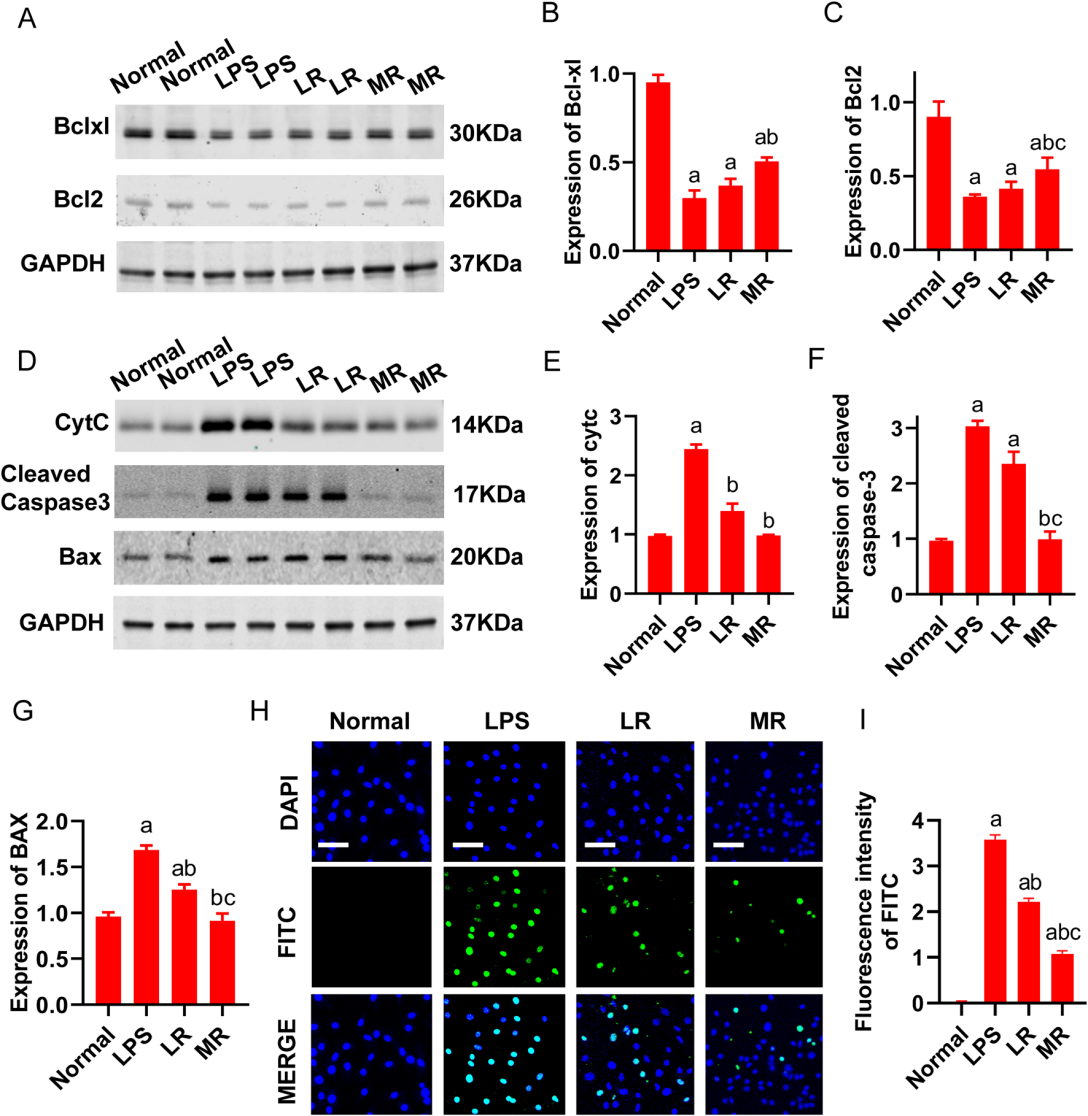
Mitochondrial morphology of cardiomyocytes after sepsis. **A**, **B** The morphology and aspect ratio of mitochondria were observed under the electron microscope (bar, 25 µm, n = 6/group). **C**, **D** The morphology and density of mitochondria in ARCMs were observed under microscope (bar, 15 µm, n = 6/group). **E** Representative images of mitochondrial morphology of H9C2 cells (bar, 10 µm, 3 independent experiments). a: P < 0.05 compared with the sham or normal group, b: P < 0.05 compared with the sepsis or LPS group, c: P < 0.05 compared with the LR group

Previous studies demonstrated that mitochondrial morphology played a decisive role in mitochondrial function, at the same time mitochondrial function also regulated mitochondrial morphology [42–44]. The images of transmission electron microscope showed that the mitochondrial membrane structure was complete, regular and dense, the cristae membranes were arranged orderly, and the length–width ratio of mitochondria was about 1.65 ± 0.42 µm in normal heart tissue. Following sepsis, the mitochondria expanded into a spherical shape, vacuoles appeared, a matrix was loose, cristae structure was fuzzy, the number of myocardial mitochondria was increased, and the length–width ratio of mitochondria was about 1.15 ± 0.20 µm. LR resuscitation could improve the structure of mitochondria, the fragmentation of mitochondria was reduced, the degree of swelling was slightly reduced, and the length–width ratio of mitochondria was about 1.26 ± 0.40 µm. Following MR resuscitation, the damage of mitochondrial structure was significantly alleviated, the mitochondrial matrix was recovered, the cristae structure was clear, and the length–width ratio of mitochondria was about 1.42 ± 0.25 µm, significantly increased compared with LR group (p < 0.05) (Fig. 5A, B).

Mitochondria of acute isolated primary adult rat cardiomyocytes (ARCMs) were stained with mitochondrial tracker. The mitochondria of acute isolated cardiomyocytes from rats were stained with Mito tracker. Confocal microscope images showed that mitochondria with long strips were evenly distributed in the cytoplasm in normal heart. The mitochondria in ARCMs presented seriously fragment after sepsis and mitochondria appeared the punctate or spherical shape. LR resuscitation could not abolish the sepsis-induced feature of mitochondria in ARCMs. While MR resuscitation could antagonize sepsis-induced mitochondrial fragmentation, and the bulk of mitochondria appeared the cord shape and distributed evenly (Fig. 5C, D). The morphological changes of mitochondria in H9C2 cells after LPS stimulation were observed by confocal microscopy. The results showed that the mitochondria in the normal cell were distributed continuously in a long column, which was consistent with the morphology and distribution of mitochondria that was observed by electron microscope in the previous heart tissue. After LPS stimulation, most mitochondria in cardiomyocytes were punctate or spherical, and the number of mitochondria were increased significantly. LR incubation did not improve the damage of mitochondrial structure. MR incubation could recover the morphology of mitochondria, and the number of mitochondria was reduced considerably (Fig. 5E). The above results suggested that MR played a protective role in mitochondrial function by inhibiting the mitochondrial division and maintaining the healthy morphology of mitochondria.

#### Effects of MR on apoptosis in cardiomyocytes after sepsis

**Fig. 6:**
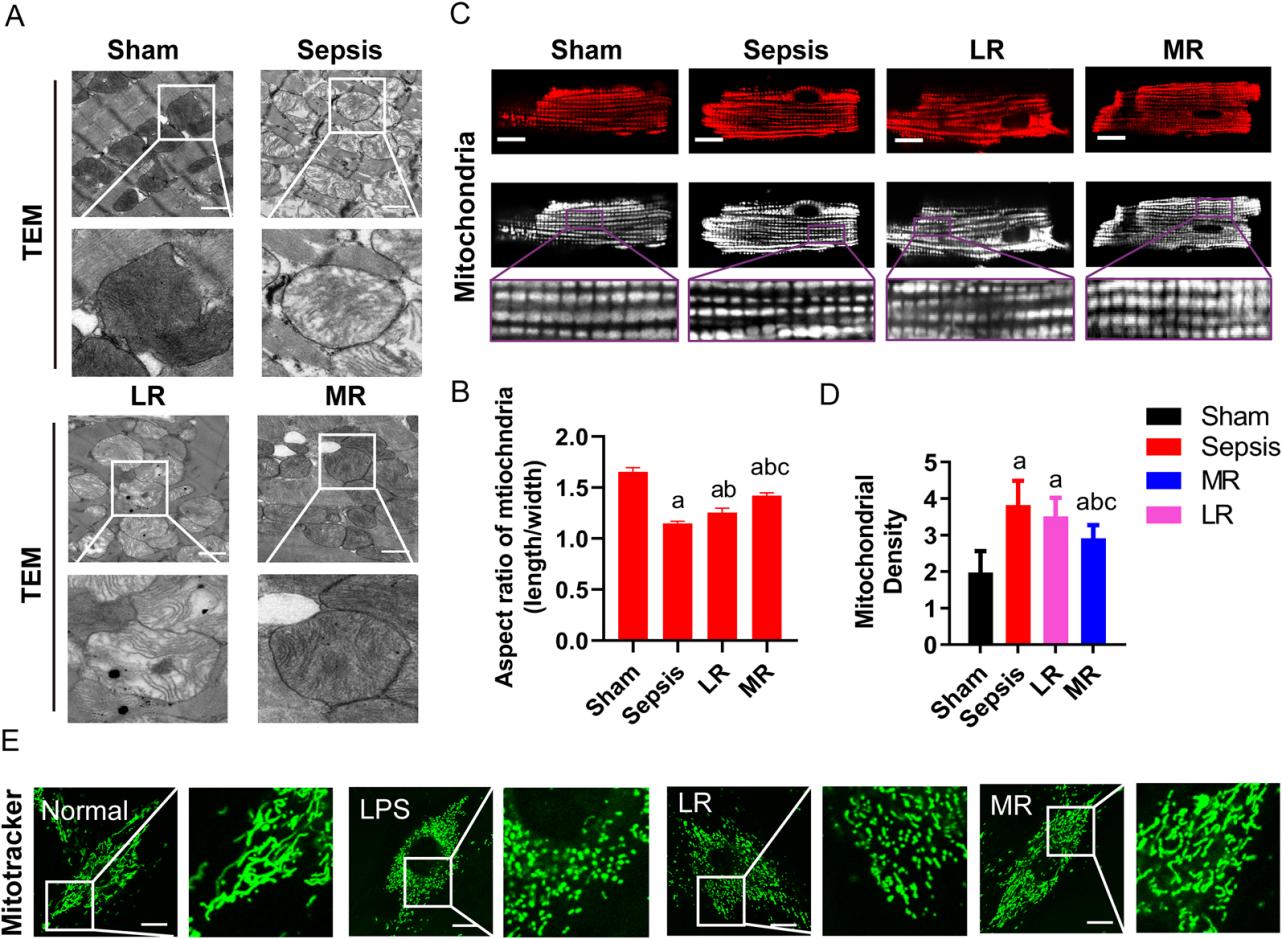
Effect of MR on H9C2 cells apoptosis. **A–C** The effect of MR on the expression of Bcl-xl and Bcl-2 proteins (3 independent experiments). **D–G** The effect of MR on Caspase-3, Cytc, and Bax protein expression (3 independent experiments). **H** Tunel was used to detect cardiomyocyte apoptotic bodies (bar, 100 µm, 3 independent experiments). **I** Quantitative analysis of apoptotic cells in H9C2 cells. a: P < 0.05, compared with normal group, b: P < 0.05, compared with LPS group, c: P < 0.05, compared with LR group

According to the proteomics results, MR infusion might alleviate the cardiomyocyte apoptosis. The Western blot results showed that the expression of anti-apoptotic proteins Bcl-xl and Bcl2 in cardiomyocytes (H9C2) were significantly reduced after the LPS stimulation, which was not reversed by LR incubation (LPS group, LR group vs normal group, p < 0.05. LPS group vs LR group, p > 0.05). The expression of Bcl-xl and Bcl2 in H9C2 cells after MR inculation was increased significantly, reaching to 37.2% and 31.9% compared with LR group, respectively (P < 0.05) (Fig. 6A–C). Moreover, the expressions of Cytochrome C (Cytc), caspase-3, and Bax in H9C2 cells were significantly increased after LPS stimulation. There was no significant change in the expression of above proteins after LR incubation. However, the expressions of Cytc, caspase-3 and Bax after MR incubation were decreased significantly to 29.8%, 58.1% and 27.1% (as compared with LR group) respectively (P < 0.05) (Fig. 6D–G). Tunel test showed that LPS stimulation led to the increase of apoptotic bodies, which were slightly decreased after LR incubation but lighten significantly after MR incubation (Fig. 6H).

### The synthesis of targeting mitochondria material containing malic acid

**Fig. 7:**
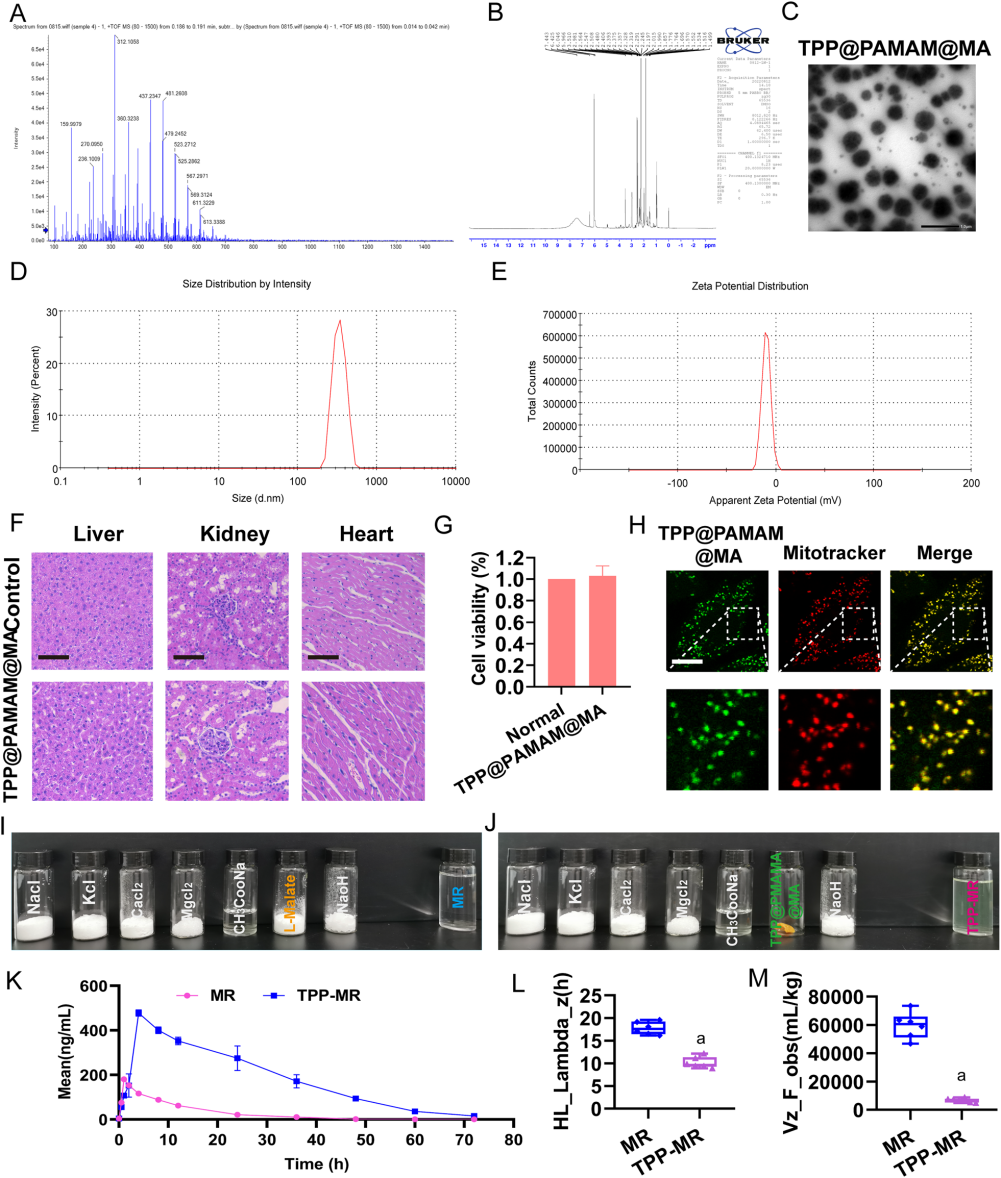
Characterization of TPP@PAMAM@MA. **A** TPP@PAMAM@MA mass spectrometry. **B** TPP@PAMAM@MA NMR determination. **C** The size of TPP@PAMAM@MA was observed under the electron microscope (bar, 1 µm). **D** Size determination of TPP@PAMAM@MA particle (3 independent experiments). **E** Potential of TPP@PAMAM@MA (3 independent experiments). **F** HE stains of heart, liver, and kidney after TPP@PAMAM@MA treatment in rats, n = 6/group. **G** Effect of TPP@PAMAM@MA on the cell viability of H9C2 cells (3 independent experiments). **H** Mitochondrial colocalization of TPP@PAMAM@MA in H9C2 cells (bar, 25 µm, 3 independent experiments). **I**, **J** MR and TPP-MR preparation

The above results showed that MR could reduce myocardial injury after sepsis by protecting myocardial mitochondria and inhibiting cell apoptosis for its malic acid. However, the entering efficiency of l-malic acid into mitochondria would be cut down due to the malate shuttle [45]. In order to improve the bioavailability of malic acid, the new material called TPP@PAMAM@MA was synthesized which embodied malic acid and targeted myocardial mitochondria. Hydrogen spectrum measurement showed that malic acid-CH2-^1^HNMR features were as δ 2.01 and δ 2.24. PAMAM –COOR ^1^H NMR features were as δ 2.01–2.58. TTP-COOH ^1^H NMR features were as 7.32–7.58 (Ar–H) (Fig. 7A). PEG ^1^H NMR features were as δ2.14–2.21 (–NHCH_2_CH_2_–) (Fig. 7B). The average particle size of the synthesized TPP@PAMAM@MA was (122.3 ± 0.8) nm, and the zeta potential was (5.7 ± 0.6) mV (Fig. 7D, E). The electron microscope pictures showed that the TPP@PAMAM@MA were spherical with regular particles (Fig. 7C).

HE staining results showed that the TPP@PAMAM@MA did not scatter in liver, kidney and heart after administration, suggesting that TPP@PAMAM@MA was safe to use in vivo, and pathological score was performed as described above [40] (Fig. 7F and Additional file 5: Fig. S5A–C). In vitro experiment was also performed to evaluate whether TPP@PAMAM@MA was toxic to cardiomyocytes. The CCK-8 assay showed that TPP@PAMAM@MA did not affect the viability of cardio H9C2 cells (Fig. 7G).

Whether TPP@PAMAM@MA could efficiently enter mitochondria was further investigated**.** Immunofluorescence results showed that TPP@PAMAM@MA could co-localize with the mitochondria, suggesting that TPP@PAMAM@MA could efficiently enter mitochondria (H9C2cells) (Fig. 7H).

The original malic acid of MR was substitute by TPP@PAMAM@MA to get the new resuscitation solution named TPP@PAMAM-MR (TPP-MR). The TPP-MR was clear and transparent, and its pH and osmotic pressure was qualified to transfusion (Fig. 7I, J). LC–MS/MS system analysis showed that TPP-MR was significantly different from MR. The peak concentration (Cmax) of TPP-MR was 472.8 ± 16.4 ng/mL, the half-life of elimination phase (HL_Lambda_z) was 10.2 ± 1.2 h, and the apparent distribution volume (Vz_F_obs) was 6724.5 ± 1486.6 mL/kg. The peak concentration (Cmax) of MR was 181.4 ± 7.9 ng/mL, the half-life of elimination phase (HL_Lambda_z) was 17.8 ± 1.4 h, and the apparent distribution volume (Vz_F_obs) was 59,557.8 ± 9257.8 mL/kg (Fig. 7L, M).

### Protect effects of TPP-MR on sepsis

**Fig. 8:**
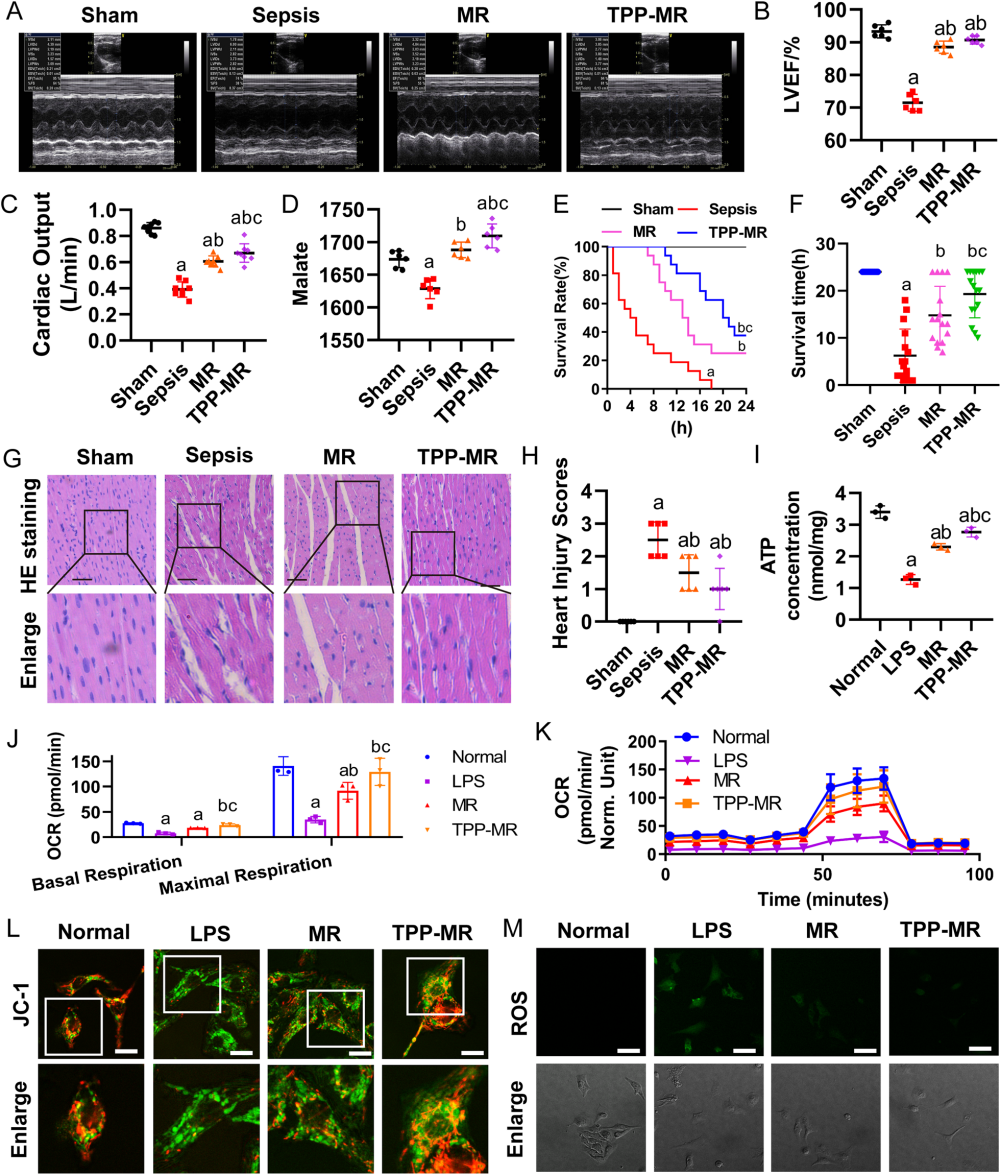
Effects of TPP-MR on myocardial injury in sepsis. **A** Cardiac function including echocardiograms (n = 6/group), **B** cardiac EF (n = 6/group) and **C** cardiac output (n = 8/group) were measured 2 h after resuscitation treatment. **D** The malic acid content in heart tissue were measured 2 h after resuscitation treatment, n = 6/group. **E–F** The effects of TPP-MR on survival rate and survival time of septic rats (n = 8/group). **G** HE stains of cardiac tissue and **H** heart injury score (bar, 100 µm, n = 6/group). **I–K** ATP and OCR detection in NRCMs (3 independent experiments). **L** The mitochondrial membrane potential of NRCMs (bar, 30 µm, 3 independent experiments). **M** ROS content in NRCMs (bar, 50 µm, 3 independent experiments). a: P < 0.05 compared with the sham or normal group, b: P < 0.05 compared with the sepsis or LPS group, c: P < 0.05 compared with the LR group

As compared with MR, TPP-MR significantly increased the LVEF, cardic output and malic acid level of septic rats (Fig. 8A–D). Moreover, although the survival time of the MR group was significantly longer than that of the sepsis group, and the 24 h survival rate of the MR group was 25%, the survival time of the TPP-MR group was prolonged to 19.3 ± 5.0 h, and the 24 h survival rate was 37.5% (Fig. 8E, F). The HE staining of cardiac pathological showed that TPP-MR further reduced the sepsis-induced interstitial edema of myocardial cells and better restored the disorder of myocardial fiber arrangement compared with MR (Fig. 8G). And perform pathological scoring as described previously [40] (Fig. 8H).

ATP content and mitochondrial respiration (OCR) were further measured at the cell level in vitro. The protective effects of TPP-MR on the LPS-induced decrease of ATP was more significant than MR (Fig. 8I). LPS stimulation significantly reduced the OCR of NRCMs, which was improved after MR treatment, while the OCR was improved considerably after TPP-MR treatment (Fig. 8J, K). The effects of TPP-MR and MR on the mitochondrial function of NRCMs after LPS stimulation were also observed. Protective effects of TPP-MR on mitochondrial membrane potential of NRCMs and ROS fluorescence intensity were stronger than the MR (Fig. 8L, M and Additional file 6: Fig. S6A, B). Besides, the same trend was observed in H9C2 cells (Additional file 6: Fig. S6C–F).

The mitochondrial structure of cardiomyocytes (H9C2 cells) stained with Mito tracker live cell mitochondrial dye was observed with the confocal microscope. Following MR treatment, the degree of mitochondrial fragmentation was decreased. After TPP-MR treatment, the fragmentation of mitochondria was further alleviated, and the mitochondria were recovered in a strip shape and distributed evenly (Additional file 6: Fig. S6G). The above results suggested that TPP-MR was superior to MR in reducing myocardial injury after sepsis.

## Discussion

Present study showed that MR resuscitation significantly prolonged the average survival time and survival rate after sepsis, improved the cardiac function, inhibited the release of inflammatory factors, reduced myocardial injury, and alleviated the damage to liver, kidney and lung. The mechanisms were related to MR enhancing the energy supply, maintaining the normal morphology of mitochondria, improving the mitochondrial function, and inhibiting the apoptosis. A material named TPP@PAMAM@MA which could target mitochondria was synthesized to enforce the efficiency of malic acid. Then we synthesized a new solution called TPP-MR by replacing the malic acid in MR with TPP@PAMAM@MA, and found that the protective effects of TPP-MR on cardiac function, cardiac mitochondrial function and structure, survival were better than MR.

LR was commonly used for resuscitation in sepsis since it could rapidly supplement the practical blood volume. However, due to its single composition and containing 28 mmol/L lactate root, LR often aggravated the accumulation of lactic acid and led to hyperlactatemia [46]. Besides, the concentration of Na^+^ was 131 mmol/L in LR, which was significantly lower than that in plasma and was easy to cause hyponatremia. Moreover, the osmotic pressure of LR was considerably lower than that of plasma, and a large amount of LR infusion easily resulted in aggravation of tissue edema. Therefore, the resuscitation effect of LR was limited. The therapeutic effect of MR was significantly better than that of LR solution. The metabolic oxygen consumption was lower and the content of HCO_3_^−^ was as high as 34 mmol/L in MR, which could effectively supplement HCO_3_^−^ and maintain the acid–base balance. In addition, the actual osmotic pressure (290 mosmol/L) of MR was comparable with that of human plasma. What’s more, MR contained a special substance called l-malic acid (330 mg/500 mL), which was a metabolic intermediate of the tricarboxylic acid cycle and directly participated in the energy metabolism of cardiac mitochondria [47, 48]. l-malic acid shuttled through the Oxoglutarate Malate carrier (OMC) channel to enter the mitochondria of cells, and generated NADH by the action of malate dehydrogenase [20, 49, 50]. Then NADH reduced oxaloacetic acid to malate, which entered the matrix through OMC on the inner membrane of mitochondria and regenerated oxaloacetic acid and NADH under the action of matrix dehydrogenase. Afterwards, the regenerated NADH entered the electronic respiratory chain to generate ATP, while oxaloacetic acid generated in the matrix was converted into aspartic acid by the action of glutamic oxaloacetic transaminase. The latter was transported out of the matrix by aspartate glutamate carrier (AGC) and then converted into oxaloacetic acid to continue shuttle to provide energy. In sepsis, the dysfunction of myocardial mitochondria disrupted oxidation of fatty acid, contributing to insufficient supply of energy. However, supplementation of malic acid by MR could significantly promote the production of ATP.

Mitochondria exhibited different changes in division and fusion to adapt to environmental changes. Under pathological conditions such as hemorrhagic shock, mitochondria of cardiomyocytes were destroyed and divided into fragmented and granular [51–53]. These changes were closely related to mitochondrial function. Present study found that after MR infusion, the mitochondrial membrane potential of cardiomyocytes was significantly improved, the ROS was reduced considerably, and the mitochondrial function was significantly improved, and the cardiomyocyte apoptosis was inhibited. In addition, infusion of MR also contributed to maintaining the normal morphology of mitochondria in septic myocardium, inhibiting mitochondrial division, and reducing mitochondrial fragmentation.

Apoptosis is an independent and orderly death of cells controlled by genes. There are three pathways of apoptosis: mitochondrial pathway, extrinsic pathway, and endoplasmic reticulum stress pathway [54]. Among them, the mitochondrial pathway is the most critical one. When cardiomyocytes were subjected to a series of changes, including oxidative phosphorylation damage, decrease in phosphokinase activity, and oxide damage, the mitochondrial membrane potential decreased, the mitochondrial membrane permeability increased, the mitochondrial permeability transition pore (mPTP) opened, and the pro-apoptotic factors such as Cytc and AIF in mitochondria were released into the cytoplasm. Afterwards, the released Cytc interacted with Apaf-1 and formed an apoptotic complex with the assistance of ATP and dATP. Then the apoptotic complex recruited and activated pro-caspase9 to assemble the Caspase9 holoenzyme, which started effector Caspase3 and caspase7, initiated caspase cascade reaction, and finally led to cell apoptosis [55–57]. We found that after MR infusion, the expression of Pro-apoptotic proteins Cytc, Caspase-3, and Bax decreased significantly. Since MR infusion increased the production of NADH as mentioned above, we hypothesized that the increased production of NADH promoted the decomposition of hydrogen peroxide which could be generated by catalyzing ROS, leading to ROS clearance. ROS could directly oxidize the cys158 and cys229 sites of Bcl-2 (a key anti-apoptotic protein) through hydrogen peroxide to inactivate Bcl-2 [58, 59]. Therefore, MR infusion indirectly activated the anti-apoptosis-related proteins, thereby inhibiting apoptosis. This hypothesis was proved by the fact that the anti-apoptotic proteins Bcl-2 and Bcl-xl of LPS-stimulated cardiomyocytes significantly increased after treatment with MR treatment in this study.

To improve the targeting and physicochemical properties of drugs, the development of targeted agents was rapid. Among them, the application of novel nano-formulations has become more extensive. Dendrimers are highly branched, monodisperse three dimensional spherical polymers. Compared with other nano-agents, dendrimers have higher transfection efficiency and stability than viral or liposome carriers, which can prolong the survival time of drugs in vivo. Jaleh et al. [60] found that dendrimer delivering active siRNA can improve the stability of siRNA in plasma and prolong the half-life of it. In addition, Palmerston et al. [61] reviewed in detail the latest progress of dendrimers as nucleic acid drug nano carriers. Thus, we wrapped l-malic acid with TPP-PEG-PAMAM, which was a material targeting mitochondria. l-malic acid in MR was replaced by TPP@PAMAM@MA to get a new solution TPP-MR. We found that the protective effect of TPP-MR on myocardial injury after sepsis was more evident than that of MR.

Although the protective effect of MR and TPP-MR on myocardial injury after sepsis was observed in this study, whether it had a defensive impact in septic patients needed further research.

## Conclusion

MR reduced sepsis-induced myocardial injury by providing energy, recovering mitochondrial morphology, and inhibiting cardiomyocyte apoptosis. The bioavailability of malic acid of MR was enhanced via synthesizing a material targeting myocardial mitochondria to encapsulate malic acid. TPP-MR has a more apparent protective effect on myocardial injury in septic rats. These findings may provide a new insight to the treatment for sepsis.

## Supplementary Information

**Additional file 1: Figure S1. (A**, **B)** Cardiac function including cardiac index and stroke index (n = 8/group).

**Additional file 2: Figure S2. (A**, **B)** The expression of glutamate transferase and aspartate transferase at 2 h after resuscitation (n = 8/group). **(C**, **D)** The expression of creatinine and urea at 2 h after resuscitation (n = 8/group).

**Additional file 3: Figure S3.** Cardiac troponin T was used to identify neonatal rat cardiomyocytes (NRCMs) (bar, 30 µm, 3 independent experiments).

**Additional file 4: Figure S4. (A)** Changes in the ATP level (3 independent experiments). **(B**, **C)** Mitochondrial maximum respiratory rate assay in H9C2 cells (3 independent experiments). **(D)** The mitochondrial membrane potential of H9C2 cells was observed by confocal microscopy (bar, 30 µm, 3 independent experiments). **(E)** The content of reactive oxygen species in H9C2 cells was observed by confocal microscopy (bar, 50 µm, 3 independent experiments).

**Additional file 5: Figure S5. (A)** Liver injury scores (n = 3/group). **(B)** Kidney injury scores (n = 3/group). **(C)** Heart injury scores (n = 3/group).

**Additional file 6: Figure S6. (A)** Red/green fluorescent (Δψm) quantitative analysis in NRCMs. **(B)** ROS fluorescence quantitative analysis in NRCMs. **(C-D)** ATP and OCR detection in H9C2 cells (3 independent experiments). **(E)** Representative images of ROS in H9C2 cells (bar, 50 µm, 3 independent experiments). **(F)** The mitochondrial membrane potential of H9C2 cells (bar, 20 µm, 3 independent experiments). **(G)** Morphological observation of mitochondria in H9C2 cells (bar, 10 µm, 3 independent experiments).

## Data Availability

The raw data of this study are available from the corresponding author on reasonable request.

## Abbreviations

LR: Ringer's solution
MR: Malate Ringer's solution
CLP: Cecal ligation and puncture
LPS: Lipopolysaccharide
MAP: Mean arterial blood pressure
DA: Dopamine
CO: Cardiac output
HR: Heart rate
CI: Cardiac index
SI: Stroke index
Bb: Hemoglobin
cTnT: Cardiac troponin T
LDH: Lactate dehydrogenase
Cytc: Cytochrome C
OMC: Oxoglutarate Malate carrier
AGC: Aspartate glutamate carrier
mPTP: Mitochondrial permeability transition pore
DDS: Drug delivery system
TPP: Triphenyl phosphine
NSH: *N*-Monohydroxy succinimide
PAMAM: Polyamidoamine
ANOVA: One-way analysis of variance
