# Correction: Effects of Malate Ringer’s solution on myocardial injury in sepsis and enforcement effects of TPP@PAMAM-MR

**DOI:** 10.1186/s12967-025-07343-z

**Published:** 2025-11-03

**Authors:** Lei Tan, Han She, Jie Zheng, Xiaoyong Peng, Ningke Guo, Bindan Zhang, Yue Sun, Chunhua Ma, Shenglian Xu, Daiqin Bao, Yuanqun Zhou, Qinghui Li, Qingxiang Mao, Liangming Liu, Yi Hu, Tao Li

**Affiliations:** 1 Department of Anesthesiology, Daping Hospital, Army Medical University, Chongqing, 400042 China; 2 State Key Laboratory of Trauma, Burns and Combined Injury, Shock and Transfusion Department, Daping Hospital, Army Medical University, Chongqing, 400042 China; 3 School of Medicine, Chongqing University, Chongqing, 400044 China

**Correction: J Transl Med 20**,** 591 (2022).**

10.1186/s12967-022-03811-y

Following publication of the original article [1], the authors reported an error in **Fig. 8M** where the ROS fluorescence intensity image of the LPS group was inaccurate. The image of the LR group in Fig. 4H overlapped with the image of the LPS group in **Fig. 8M**.

The incorrect version of **Fig. 8** was:


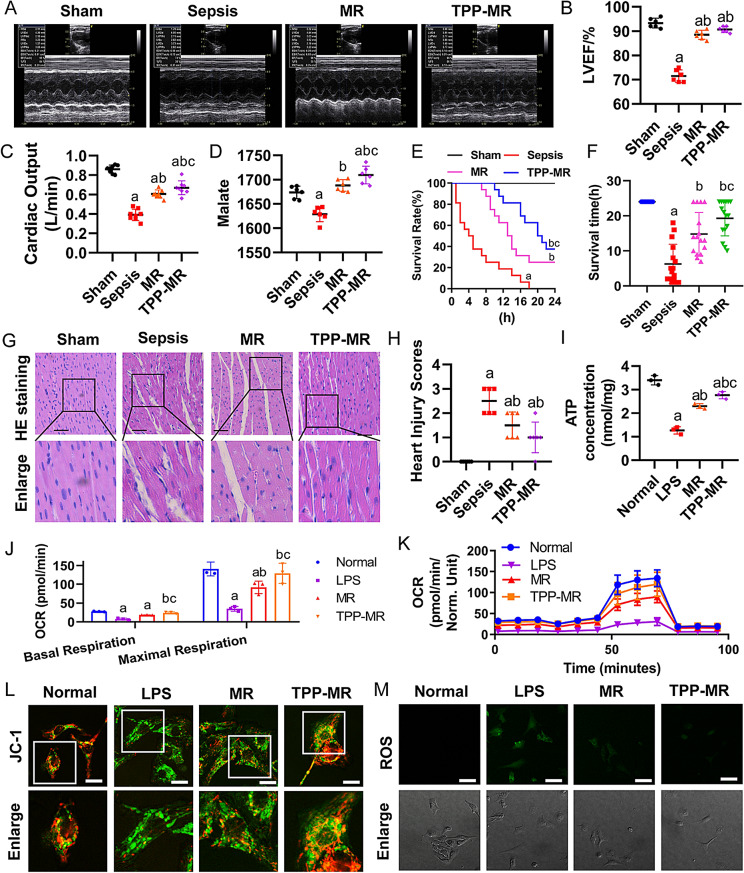


The correct **Fig. 8** is:


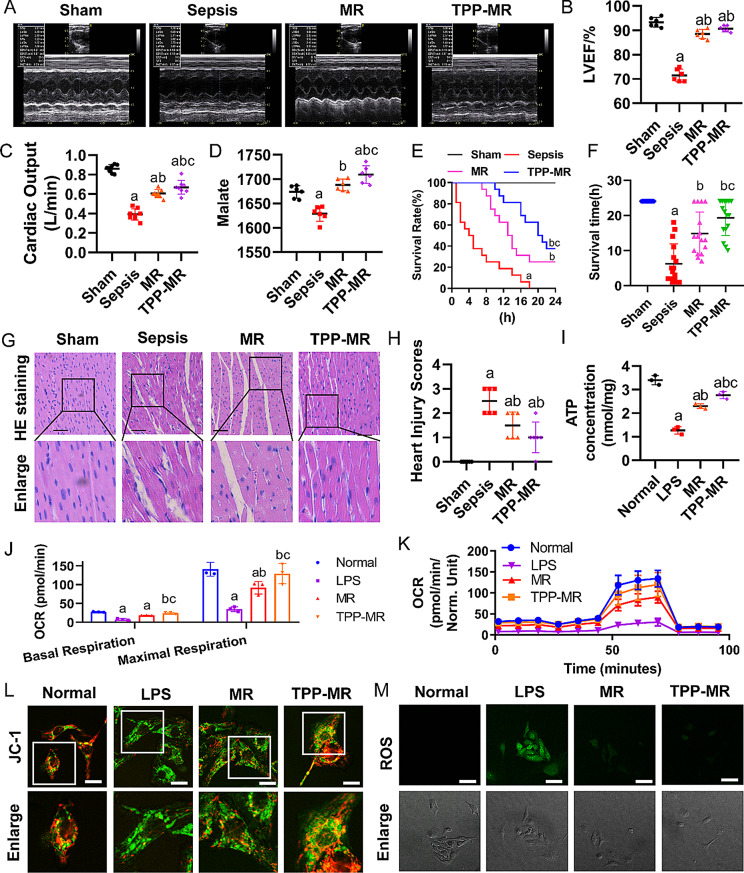


The original article [1] has been updated.

## References

[1] Tan L, She H, Zheng J, et al. Effects of malate ringer’s solution on myocardial injury in sepsis and enforcement effects of TPP@PAMAM-MR. J Transl Med. 2022;20:591. 10.1186/s12967-022-03811-y. 36514103 10.1186/s12967-022-03811-y PMC9746071

