# Supplementary figures and images for: Effects of Malate Ringer's solution on myocardial injury in sepsis and enforcement effects of TPP@PAMAM-MR
Source: J Transl Med. 2022 Dec 13;20:591. doi: 10.1186/s12967-022-03811-y (PMC9746071; doi:10.1186/s12967-022-03811-y)

A

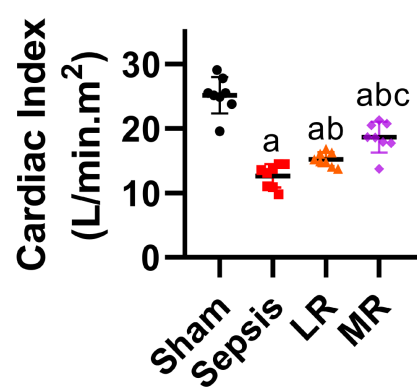

B

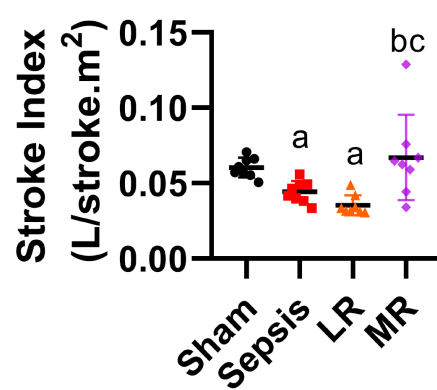

Supplement: Supplementary file 1 — Additional file 1: Figure S1. (A, B) Cardiac function including cardiac index and stroke index (n = 8/group). [file 12967_2022_3811_MOESM1_ESM.pdf]

A

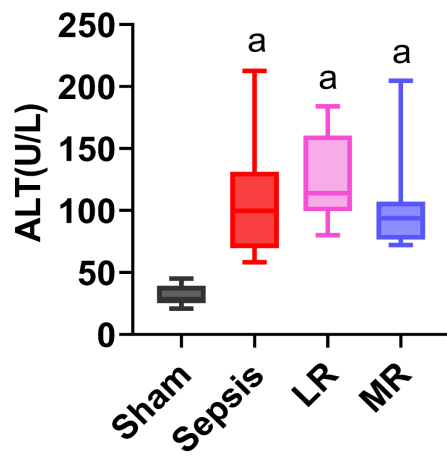

B

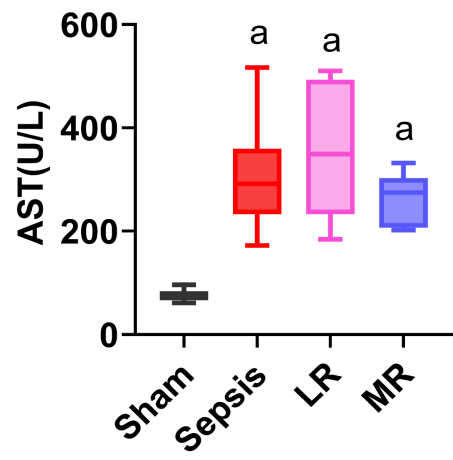

C

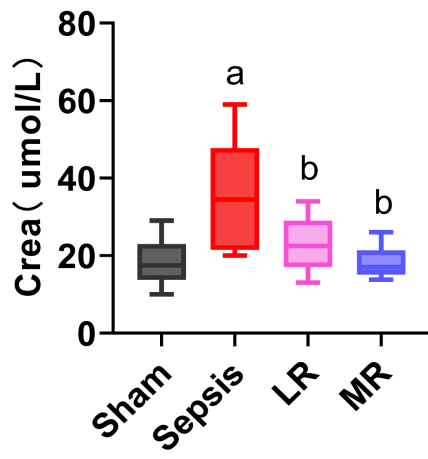

D

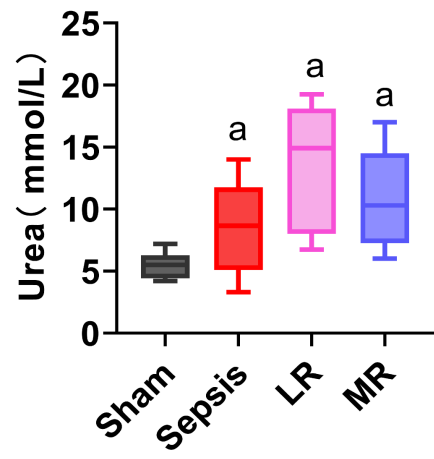

Supplement: Supplementary file 2 — Additional file 2: Figure S2. (A, B) The expression of glutamate transferase and aspartate transferase at 2 h after resuscitation (n = 8/group). (C, D) The expression of creatinine and urea at 2 h after resuscitation (n = 8/group). [file 12967_2022_3811_MOESM2_ESM.pdf]

Cardiac Troponin T (cTnT)

NRCM

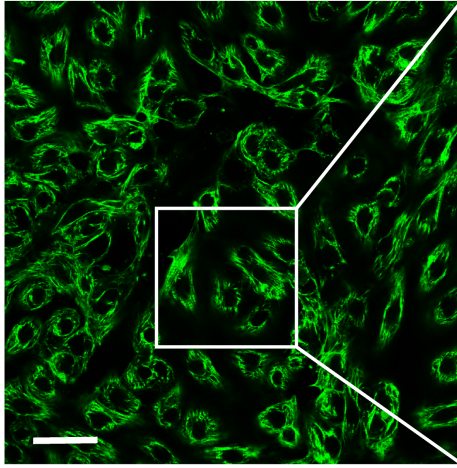

Enlarge

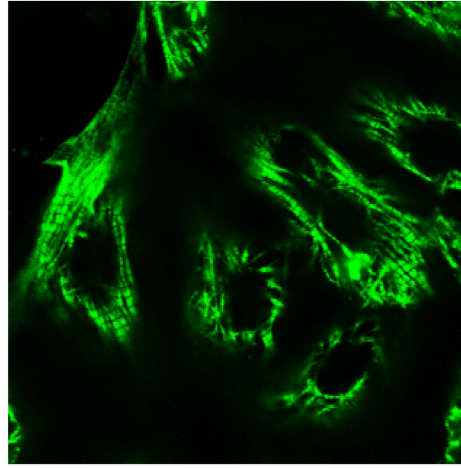

Supplement: Supplementary file 3 — Additional file 3: Figure S3. Cardiac troponin T was used to identify neonatal rat cardiomyocytes (NRCMs) (bar, 30 µm, 3 independent experiments). [file 12967_2022_3811_MOESM3_ESM.pdf]

A

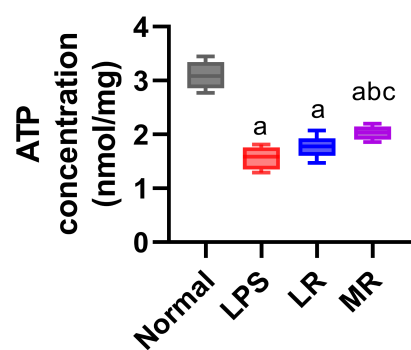

B

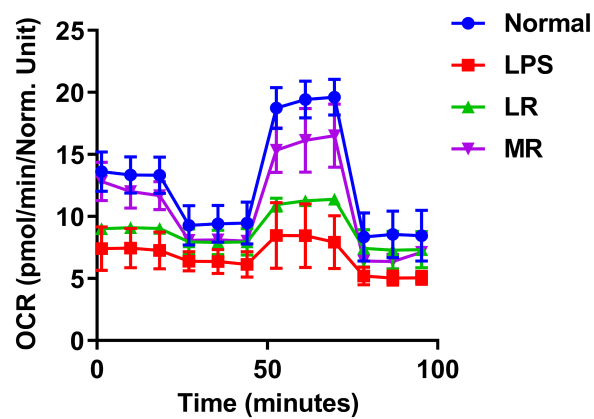

D

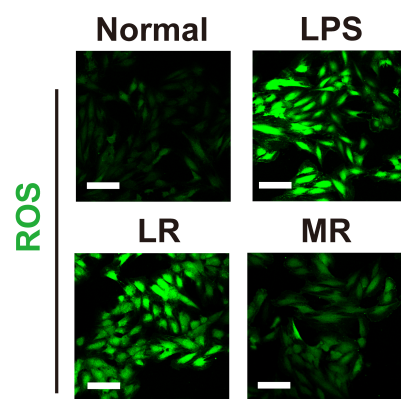

C

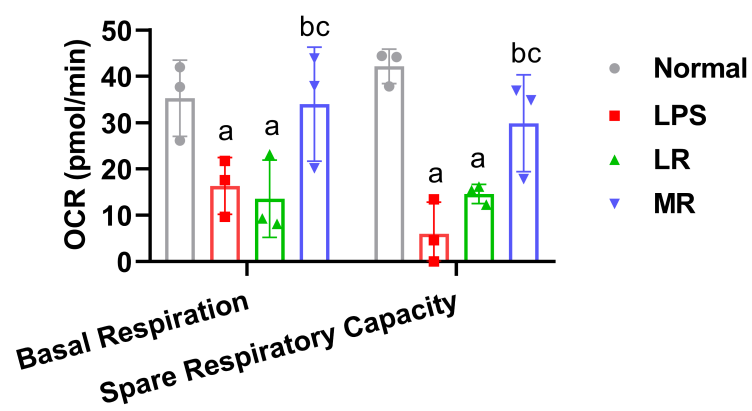

E

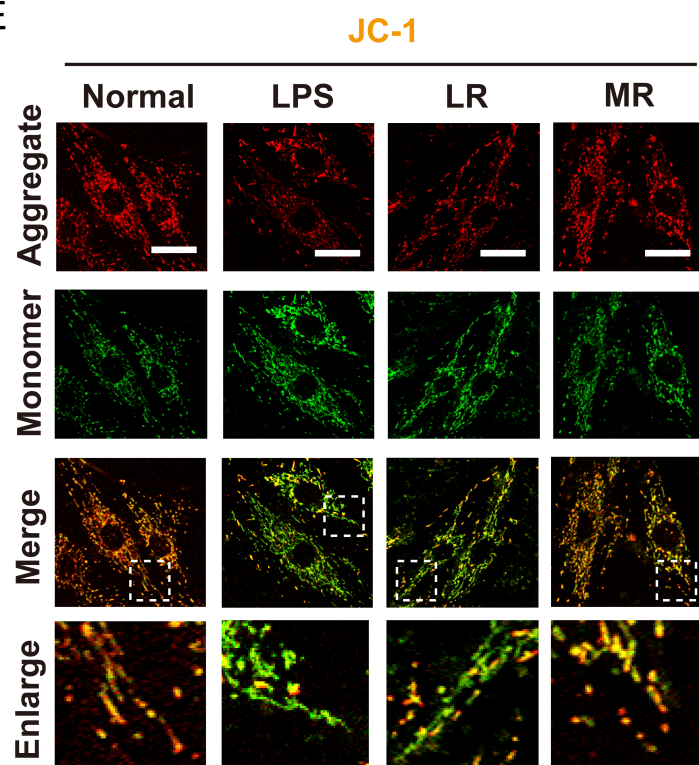

Supplement: Supplementary file 4 — Additional file 4: Figure S4. (A) Changes in the ATP level (3 independent experiments). (B, C) Mitochondrial maximum respiratory rate assay in H9C2 cells (3 independent experiments). (D) The mitochondrial membrane potential of H9C2 cells was observed by confocal microscopy (bar, 30 µm, 3 independent experiments). (E) The content of reactive oxygen species in H9C2 cells was observed by confocal microscopy (bar, 50 µm, 3 independent experiments). [file 12967_2022_3811_MOESM4_ESM.pdf]

A

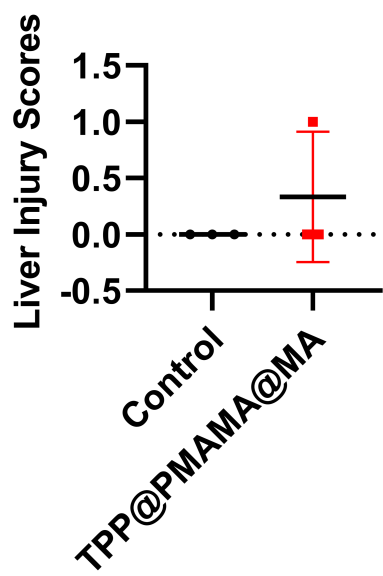

B

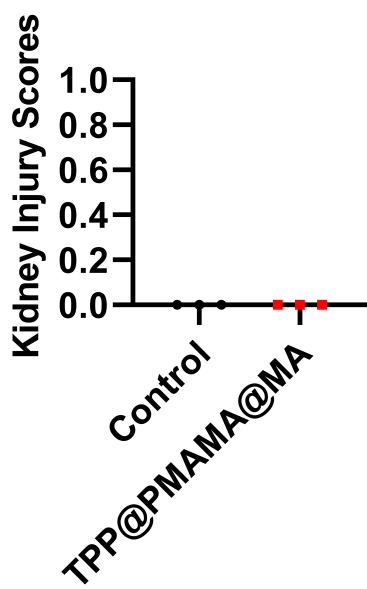

C

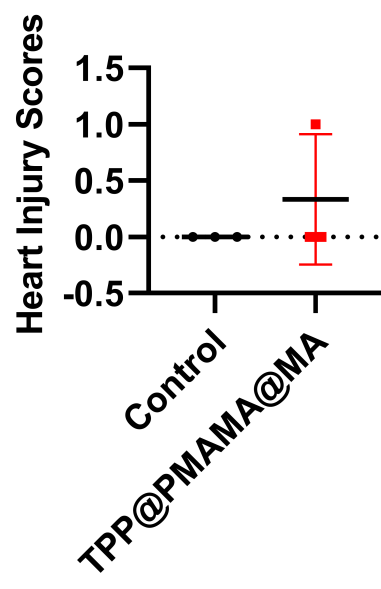

Supplement: Supplementary file 5 — Additional file 5: Figure S5. (A) Liver injury scores (n = 3/group). (B) Kidney injury scores (n = 3/group). (C) Heart injury scores (n = 3/group). [file 12967_2022_3811_MOESM5_ESM.pdf]

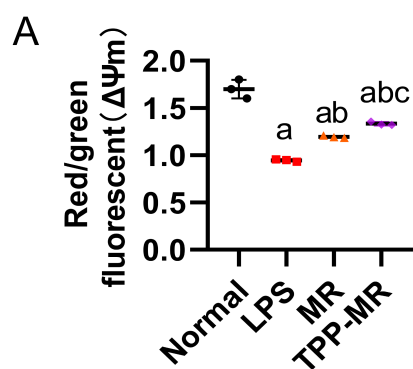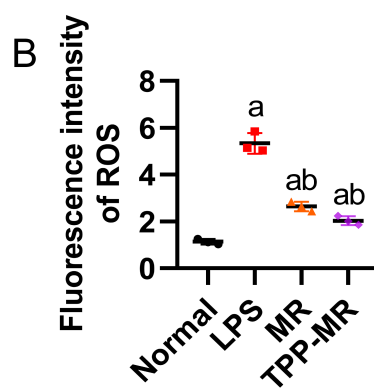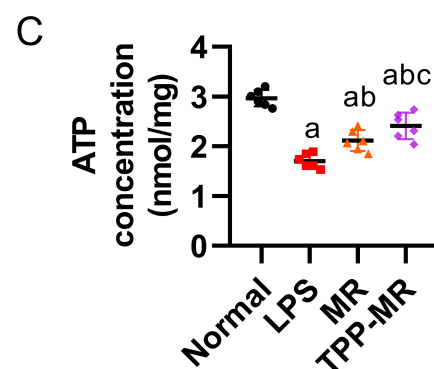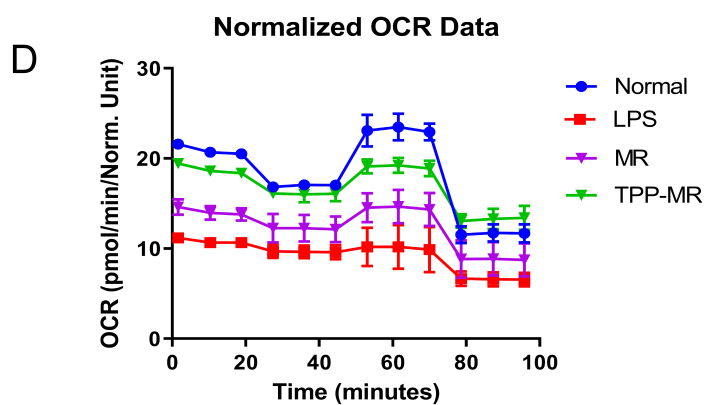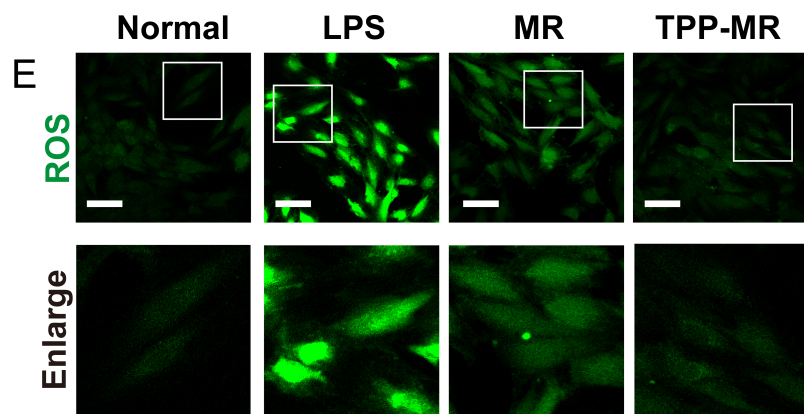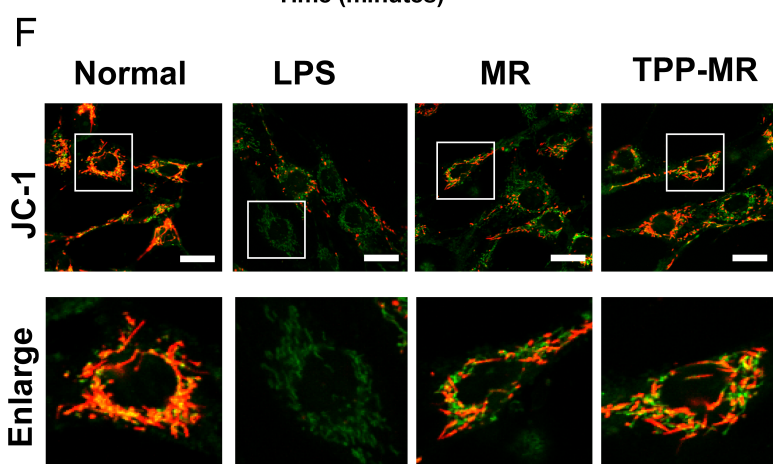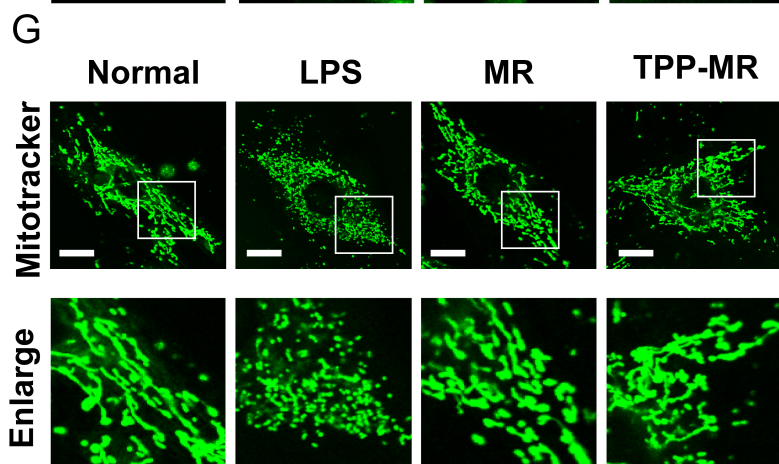

Supplement: Supplementary file 6 — Additional file 6: Figure S6. (A) Red/green fluorescent (Δψm) quantitative analysis in NRCMs. (B) ROS fluorescence quantitative analysis in NRCMs. (C-D) ATP and OCR detection in H9C2 cells (3 independent experiments). (E) Representative images of ROS in H9C2 cells (bar, 50 µm, 3 independent experiments). (F) The mitochondrial membrane potential of H9C2 cells (bar, 20 µm, 3 independent experiments). (G) Morphological observation of mitochondria in H9C2 cells (bar, 10 µm, 3 independent experiments). [file 12967_2022_3811_MOESM6_ESM.pdf]
